# Traumatic brain injury alters the effects of class II invariant peptide (CLIP) antagonism on chronic meningeal CLIP + B cells, neuropathology, and neurobehavioral impairment in 5xFAD mice

**DOI:** 10.1186/s12974-024-03146-z

**Published:** 2024-06-27

**Authors:** Jaclyn Iannucci, Reagan Dominy, Shreya Bandopadhyay, E. Madison Arthur, Brenda Noarbe, Amandine Jullienne, Margret Krkasharyan, Richard P. Tobin, Aleksandr Pereverzev, Samantha Beevers, Lavanya Venkatasamy, Karienn A. Souza, Daniel C. Jupiter, Alan Dabney, Andre Obenaus, M. Karen Newell-Rogers, Lee A. Shapiro

**Affiliations:** 1 Department of Neuroscience and Experimental Therapeutics, College of Medicine, Texas A&M University, Bryan, TX USA; 2 Department of Medical Physiology, College of Medicine, Texas A&M University, Bryan, TX USA; 3 Division of Biomedical Sciences, University of California Riverside, Riverside, CA USA; 4 Department of Surgery, Division of Surgical Oncology, University of Colorado Anschutz Medical Campus, Denver, CO USA; 5 Department of Biostatistics and Data Science, Department of Orthopaedics and Rehabilitation, The University of Texas Medical Branch, Galveston, TX USA; 6 Department of Statistics, College of Arts & Sciences, Texas A&M University, College Station, TX USA

**Keywords:** Alzheimer’s disease, Fluid percussion injury, Depression, Neurobehavior, Neuroinflammation, Cerebrovascular, CD74, MHCII, Innate immune, Adaptive immune

## Abstract

**Background:**

Traumatic brain injury (TBI) is a significant risk factor for Alzheimer’s disease (AD), and accumulating evidence supports a role for adaptive immune B and T cells in both TBI and AD pathogenesis. We previously identified B cell and major histocompatibility complex class II (MHCII)-associated invariant chain peptide (CLIP)-positive B cell expansion after TBI. We also showed that antagonizing CLIP binding to the antigen presenting groove of MHCII after TBI acutely reduced CLIP + splenic B cells and was neuroprotective. The current study investigated the chronic effects of antagonizing CLIP in the 5xFAD Alzheimer’s mouse model, with and without TBI.

**Methods:**

12-week-old male wild type (WT) and 5xFAD mice were administered either CLIP antagonist peptide (CAP) or vehicle, once at 30 min after either sham or a lateral fluid percussion injury (FPI). Analyses included flow cytometric analysis of immune cells in dural meninges and spleen, histopathological analysis of the brain, magnetic resonance diffusion tensor imaging, cerebrovascular analysis, and assessment of motor and neurobehavioral function over the ensuing 6 months.

**Results:**

9-month-old 5xFAD mice had significantly more CLIP + B cells in the meninges compared to age-matched WT mice. A one-time treatment with CAP significantly reduced this population in 5xFAD mice. Importantly, CAP also improved some of the immune, histopathological, and neurobehavioral impairments in 5xFAD mice over the ensuing six months. Although FPI did not further elevate meningeal CLIP + B cells, it did negate the ability of CAP to reduce meningeal CLIP + B cells in the 5xFAD mice. FPI at 3 months of age exacerbated some aspects of AD pathology in 5xFAD mice, including further reducing hippocampal neurogenesis, increasing plaque deposition in CA3, altering microgliosis, and disrupting the cerebrovascular structure. CAP treatment after injury ameliorated some but not all of these FPI effects.

**Supplementary Information:**

The online version contains supplementary material available at 10.1186/s12974-024-03146-z.

## Background

Traumatic brain injury (TBI) affects over 2–3 million people annually in the United Sates and over 50 million worldwide [1, 2]. TBI is a significant risk factor for Alzheimer’s disease (AD), the most common form of dementia, increasing the risk for AD by 2.3–4.5-fold. Of those who develop AD after TBI, the data suggest a lower average age of disease onset [3, 4]. AD is characterized by progressive memory loss and cognitive dysfunction, which are also common features of TBI [5, 6]. Considering the increased risk for AD in the millions of people who suffer a TBI each year, the 23 million Americans over 40 with a reported history of TBI with loss of consciousness [7], and the estimated 6.5 million Americans with AD in 2022 [8], it is vital to identify novel pathogenic mechanisms and therapeutic targets.

In both TBI and AD, innate immune and neuroimmune activation have been reported in clinical and preclinical studies [9–13]. As part of the innate immune response, glial cells and other immune cells are activated by, and release, pro-inflammatory cytokines and chemokines. The TBI-induced inflammatory response can result in a transition to an antigen-specific adaptive immune response [14–16], as evidenced by activation of immune cells and antibody fragments of brain specific proteins [9, 17]. An analogous adaptive immune response has also been identified in both clinical and preclinical studies of AD [18–20]. It is possible that adaptive immune components might link TBI and AD.

Accumulating evidence supports a pathogenic role for adaptive immune cell subsets in TBI and AD, including B and T cell subsets [14, 21–24]. TBI pathology is augmented by activation of inflammatory T cells, including expansion of CD8 + T cells and loss of regulatory T cells [16, 25]. In AD, T cells infiltrating into the CNS in both clinical and preclinical models is also associated with worse outcomes [26–29]. The activation of T cells occurs in response to antigen presentation by professional antigen presenting cells, including B cells.

TBI induces an increase in peripheral B cells after injury [14, 30, 31], including altered B cell signatures in the meninges [32]. Exogenous administration of purified naïve B cells is beneficial to TBI pathogenesis [33]. In AD patients, reduced peripheral B cell subsets have been detected [22, 23], and mature B cells have been identified in the AD brain [21, 34]. In a preclinical study using three genetic AD mouse models, therapeutic depletion of B cells was protective against AD-associated pathology [21]. Therefore, activation and expansion of B cell subsets may represent a shared feature in TBI and AD that can be targeted to improve post-traumatic outcomes and alter the course of AD pathogenesis. This notion is further supported by the finding that depression and cognitive impairment have been linked with altered B cell subsets [21, 35–37], and are common clinical syndromes associated with both TBI and AD [38–42].

Most TBIs, including preclinical models, are closed head, sterile injuries. Thus, adaptive immune activation after TBI may be an auto-immune response to TBI-generated self-antigens. Major Histocompatibility Complex *cl*ass II-associated *i*nvariant *p*eptide (CLIP) occupies the antigen binding groove of MHCII and plays a major mechanistic role in self-antigen presentation [43]. Previous studies have characterized a CLIP antagonist peptide (CAP) that preferentially targets CLIP + B cells [43–45]. Treatment with CAP after the fluid percussion injury (FPI) model of TBI was neuroprotective, anti-inflammatory, anti-neuroinflammatory, and acutely reduced splenic CLIP + B cells [14]. Given the involvement of B cells in both TBI and AD, as well as their role in activating T cells, we hypothesized that antagonizing CLIP would improve AD pathogenesis and ameliorate FPI-induced changes to the AD phenotype in the 5xFAD mice. To test this hypothesis, a single dose of CAP was administered at 30 min after an FPI or sham injury in male 5xFAD mice. Immune outcomes, neuroinflammation, AD-associated neuropathology, and motor and chronic neurobehavioral outcomes were assessed over the ensuing 6 months.

## Results

### Pathological assessments

#### A single dose of CAP selectively and chronically reduces meningeal CLIP + B cells

**Fig. 1:**
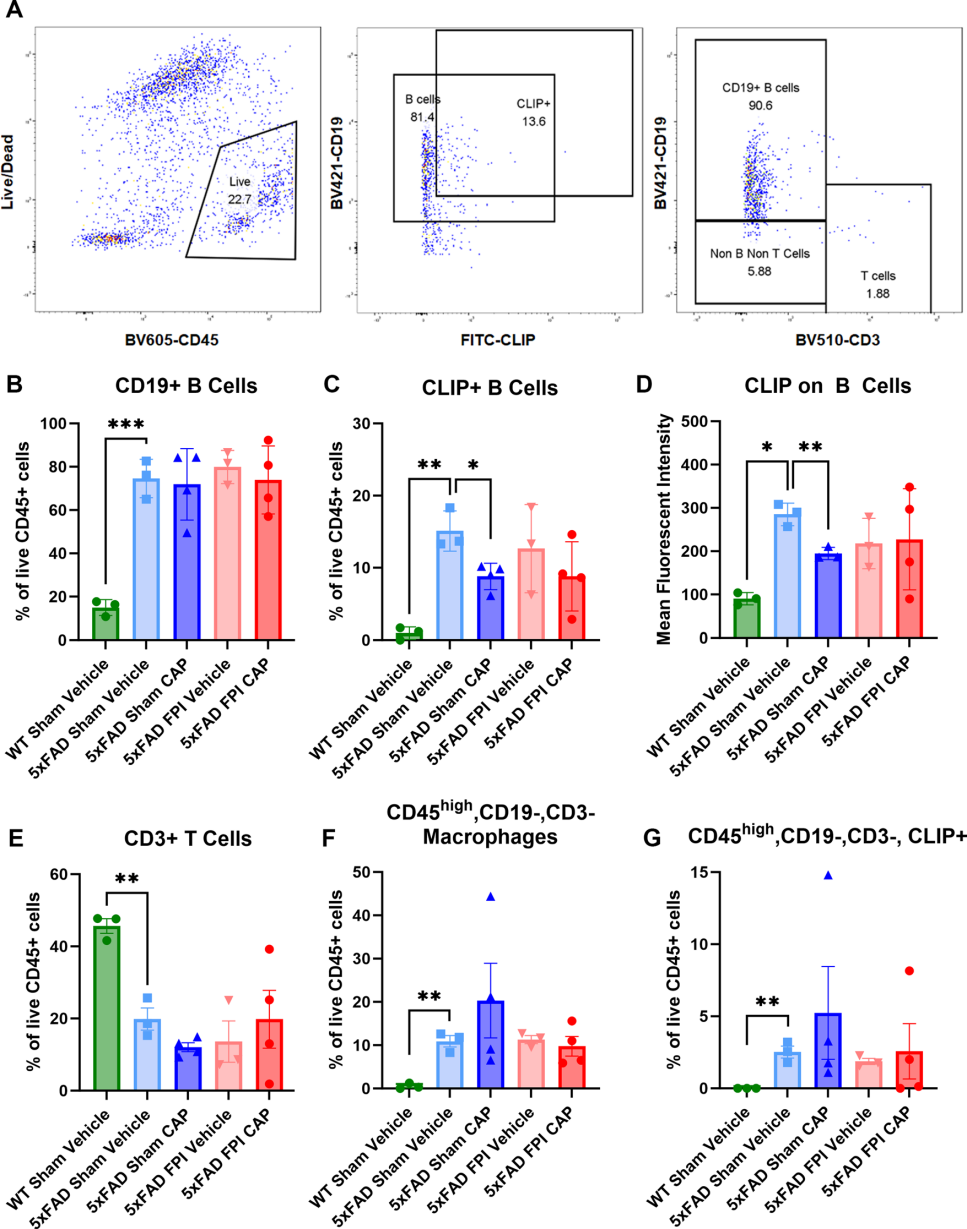
**CAP treatment results in a selective and chronic reduction in meningeal CLIP + B cells in 5xFAD mice, but not after FPI.** In (**A**), the gating strategy used to identify B and CLIP + B cells, T Cells, and macrophages. In (**B**), 5xFAD mice have significantly elevated B cells that are not changed by CAP or by FPI. In (**C**) and (**D**), CLIP + B cells and CLIP expression on B cells are significantly elevated in 5xFAD mice compared to WT mice, and significantly reduced by CAP treatment given 6 months prior. The CAP treatment did not significantly reduce CLIP + B cells in the FPI mice (**C**, **D**). In (**E**), there was a significant decrease in CD3 + T cells in the meninges in 5xFAD mice that was not affected by CAP treatment or FPI. In (**F**), CD45_high_/CD19-/CD3- macrophages are significantly elevated in 5xFAD mice, as is CLIP expression in these cells (**G**). CAP treatment did not have any significant effect on these CD45_high_/CD19-/CD3- macrophages. Data are represented as Mean ± SEM; *n* = 3 for vehicle groups, *n* = 4 for CAP groups; *p < 0.05, **p < 0.01, **p < 0.001

Flow cytometric analysis of the meninges and underlying superior sagittal sinus and meningeal lymphatic (hereto referred to as meninges) [46] was performed. The results showed that 9-month-old 5xFAD Sham + Vehicle (Veh) mice exhibited a significantly greater percent of B cells (*p* < 0.001) and CLIP + B cells (*P* < 0.01), and a higher expression level of CLIP on B cells (*p* < 0.05), compared to age-matched WT Sham + Veh mice (Fig. 1B-D). In the 5xFAD mice, CAP given at 12 weeks of age had no effect on the percent of meningeal B cells, but significantly reduced the percent of CLIP + B cells (*p* < 0.05) and the cell surface expression of CLIP on B cells (*p* < 0.01), compared to age-matched 9-month-old 5xFAD Sham + Veh mice (Fig. 1B-D). Conversely, in the 12-week-old 5xFAD mice that received CAP at 30 min after FPI, CAP treatment failed to significantly reduce the elevated meningeal CLIP + B cells (Fig. 1B-D). Therefore, FPI negated the ability of CAP to chronically reduce meningeal CLIP + B cells in 5xFAD mice.

Further examination of meningeal immune cell types showed that the 5xFAD Sham + Veh mice exhibited significantly reduced CD3 + T cells (*p* < 0.01; Fig. 1E), and significantly increased macrophages (*p* < 0.01), including CLIP + macrophages (*p* < 0.01), compared to WT Sham + Veh mice (Fig. 1F-G). Neither CAP treatment nor FPI had a significant effect on these immune subsets in the 5xFAD mice (Fig. 1E-G). Thus, CAP specifically depleted meningeal CLIP + B Cells in 5xFAD + Veh mice, but not 5xFAD FPI mice.

**Fig. 2:**
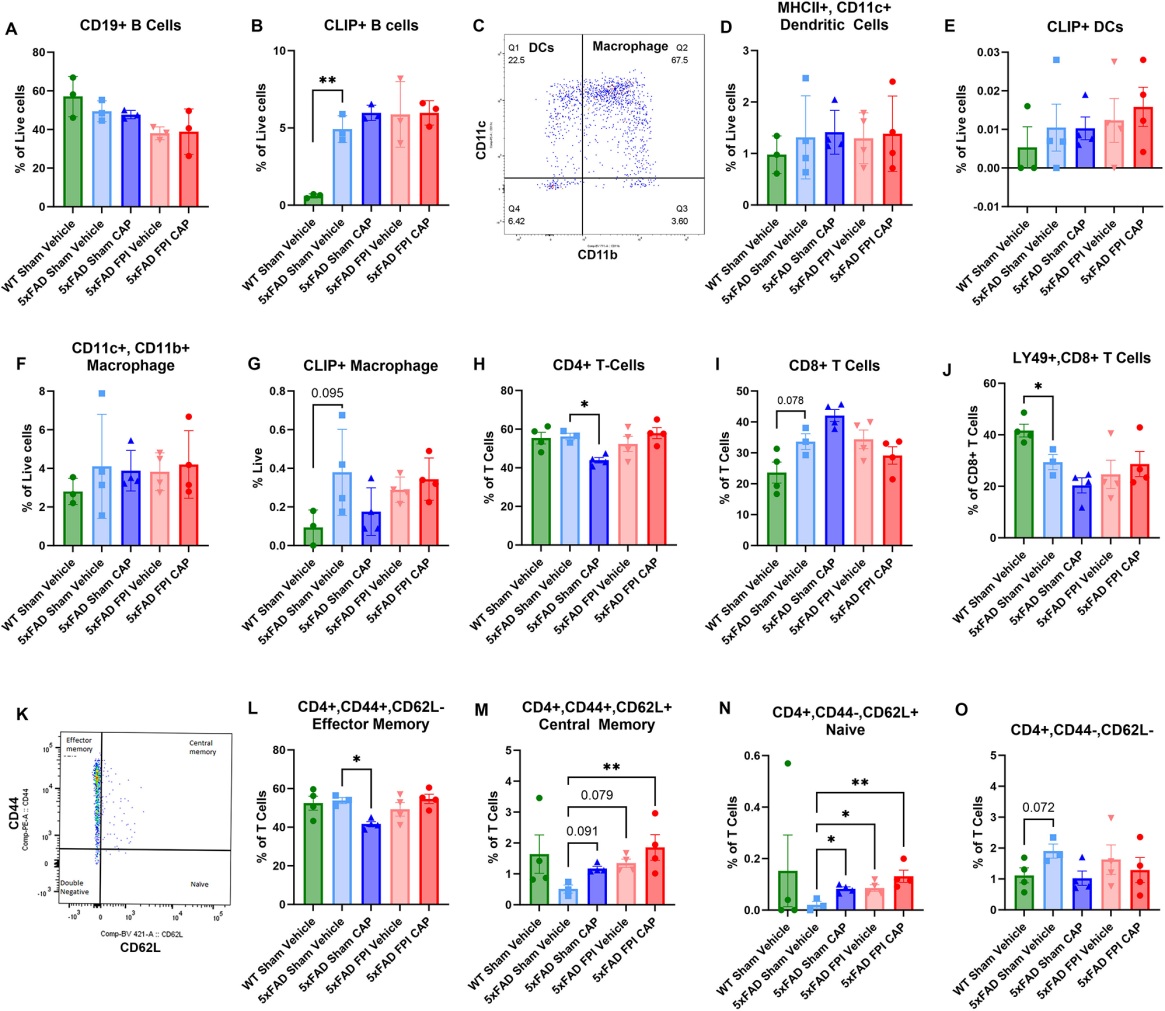
**Effects of CAP and FPI on splenocytes in 5xFAD mice**. In (**A**), there are no significant differences in splenic B cells. In (**B**), CLIP + B cells are significantly elevated in the spleen of 5xFAD mice compared to WT, but this elevation is not affected by CAP treatment or FPI. In (**C**), the gating for identifying dendritic cells and macrophages. In (**D**) **a**nd (**E**), no significant differences were observed for dendritic cells (DCs) or CLIP + DCs. In (**E**) and (**F**), although there were no significant differences in splenic macrophages, there was a trend towards an elevation of CLIP + macrophages in the 5xFAD mice that was not affected by CAP or FPI. Examination of CD4+ (**H**) and CD8+ (**I**) T cells revealed that CAP treatment significantly reduced CD4 + T cells in 5xFAD mice, whereas the CD8 + T cells exhibited a trend towards an increase in the 5xFAD mice that was not influenced by CAP. Conversely, Ly49 + CD8 cells are significantly decreased in the 5xFAD mice (**J)**. Analysis of memory cell subsets (**K-O)** revealed that central memory CD4+ (**M**) and naïve CD4 + cells (**N**) were reduced in 5xFAD mice. CAP treatment, FPI, and FPI + CAP treatment all increased these populations of cells, suggesting that CAP and FPI could be restoring the ability of T cells to activate in the condition of chronic inflammation induced by the genotypic effects of the 5xFAD mice. Data represented as Mean ± SEM; *n* = 3–4 per group; *p < 0.05, **p < 0.01

Peripheral immune cells from the spleens were also examined from these mice. The results show that the percent of CLIP + B Cells was significantly increased in 5xFAD Sham + Veh mice compared to WT Sham + Veh mice (*p* < 0.01) (Fig. 2B). Unlike the meninges, there was no chronic effect of CAP treatment on the percentage of splenic CLIP + B cells (Fig. 2B). Analysis of MHCII+/CD11c + dendritic cells (DCs), CLIP + DCs, CD11c+/Cd11b + macrophages, and CLIP + macrophages in the spleen revealed no significant differences (Fig. 2D-G).

T cell analysis showed no significant differences in the percentage of CD4 + T cells, although there was a trend towards an increased percentage of CD8 + T cells (*p* = 0.078) in 5xFAD Sham + Veh compared to WT Sham + Veh (Fig. 2H-I). Compared to WT Sham + Veh mice, 5xFAD Sham + Veh mice had a significantly decreased percentage of CD8+/Ly49 + T cells (*p* < 0.05), a population of CD8 + T cells recently described as regulators of autoimmunity [47, 48] (Fig. 2J). CAP treatment significantly reduced CD4 + T cells in the 5xFAD mice (*p* < 0.05). No such effect was observed for CD8 + T cells or CD8+/Ly49 + T cells in either CAP or FPI mice. A notable finding was that both CAP treatment and FPI increased CD44+/CD62L + central memory CD4 + T cells, (*p* = 0.091, *p* = 0.079, respectively), and in 5xFAD FPI + CAP mice, the increase in these central memory CD4+ T cells was significant (*p* < 0.01). CAP treatment and FPI significantly increased CD44-/CD62L + naïve CD4 + T cells, compared to 5xFAD Sham + Veh mice (*p* < 0.05, *p* < 0.05, respectively) (Fig. 2K-O).

#### Increased CD74 + cells in the hippocampus of 5xFAD mice is reduced by CAP

**Fig. 3:**
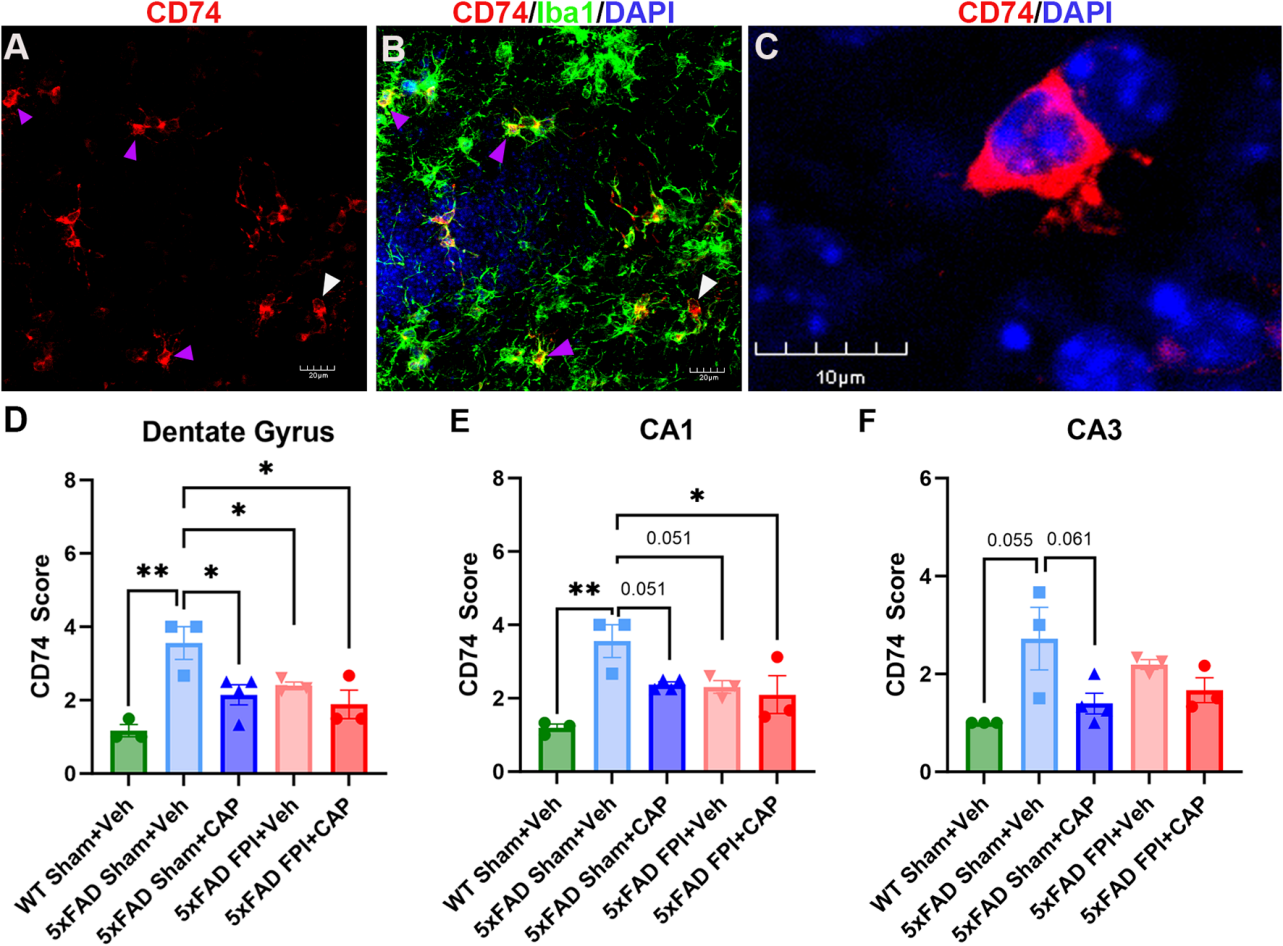
**Significantly elevated CD74 + cells in the hippocampus of 5xFAD mice are reduced by CAP treatment and FPI**. In (**A**-**C**), confocal micrographs of CD74 and CD74/Iba1 + cells in the hippocampus. The majority of CD74+ cells co-labeled with Iba1 (purple arrowheads), while a minority of cells were not co-labeled (white arrowheads), indicating that most, but not all, CD74+ cells are likely macrophage/microglia. Analysis of CD74 cells in the hippocampus revealed significant elevations in the DG (**D**), CA1 (**E**) and a trend towards elevation in CA3 (**F**) in 5xFAD mice compared to WT mice. CAP treatment significantly decreased these cells in the dentate gyrus (**D**) and CA1 (**E**) and exhibited a trend towards a reduction in CA3 (**F**). Scale bars in A-C = 30 μm, and 10 μm in D. Data are represented as Mean ± SEM; *n* = 3 for all groups; *p < 0.05, **p < 0.01

CD74 is a key immune regulator that is involved in both the innate and adaptive immune response and is often expressed at increased levels on multiple cell types under conditions of inflammation [49, 50]. CLIP is a proteolytic cleavage product of CD74 [49–51] that is produced in the lysosome [49]. CD74 has been implicated in AD, and CD74 as well as its cell-surface ligand, MIF, have been identified as potential therapeutic targets for AD and TBI [14, 30, 52–56]. There are scant reports of CD74 + cells in the brain, although in WT rodents the CD74 + cells appear to be largely restricted to the ependymal zones and choroid plexus [57]. Consistent with these data, our analysis of CD74 + cells in the brains of the WT Sham + Veh mice confirmed that the CD74 + cells were largely restricted to the ependymal and choroid plexus regions (not shown). Conversely, in the 5xFAD mice, CD74 + cells were prominently observed in the ependymal, choroidal, and parenchymal regions, including the neocortex and archicortex. Double-labeling of the CD74 + cells in the hippocampus showed that none of the CD74 + cells were GFAP+ (not shown), whereas most were either macrophages or microglia, based on double-labeling for anti-Iba1(Fig. 3A-B). The CD74 labeling appeared to be primarily around the perikaryal cytoplasm and the proximal processes (Fig. 3C).

Semi-quantitative analysis of the CD74 + cells in the hippocampus demonstrated that they were significantly increased in CA1 (*p* < 0.01) and the DG (*p* < 0.01) of the 5xFAD Sham + Veh mice compared to WT Sham + Veh. There was also a trend towards an increase in area CA3 (*p* = 0.055), indicating a robust genotype effect on the presence of hippocampal CD74 + cells (Fig. 3D-F). CAP treatment significantly reduced the number of CD74 + cells in 5xFAD sham mice in the DG (*p* < 0.05) and trends in the reduction of these cells was observed in CA1 (*p* = 0.051) and CA3 (*p* = 0.061) (Fig. 3D-F). The ability of CAP to reduce CD74 + cells in the hippocampus of 5xFAD mice is consistent with our previous study showing that CAP reduces CD74 + splenocytes [14]. The 5xFAD FPI + Veh mice exhibited a significant reduction in CD74 + cells in the DG (*p* < 0.05) and a trend in CA1 (*p* = 0.051) compared to 5xFAD Sham + Veh mice. The 5xFAD FPI + CAP mice had significantly less CD74 + cells in CA1 (*p* < 0.05) and the DG (*p* < 0.05).

#### CAP mitigates FPI-induced alterations in hippocampal CA3 plaque deposition in 5xFAD mice

**Fig. 4:**
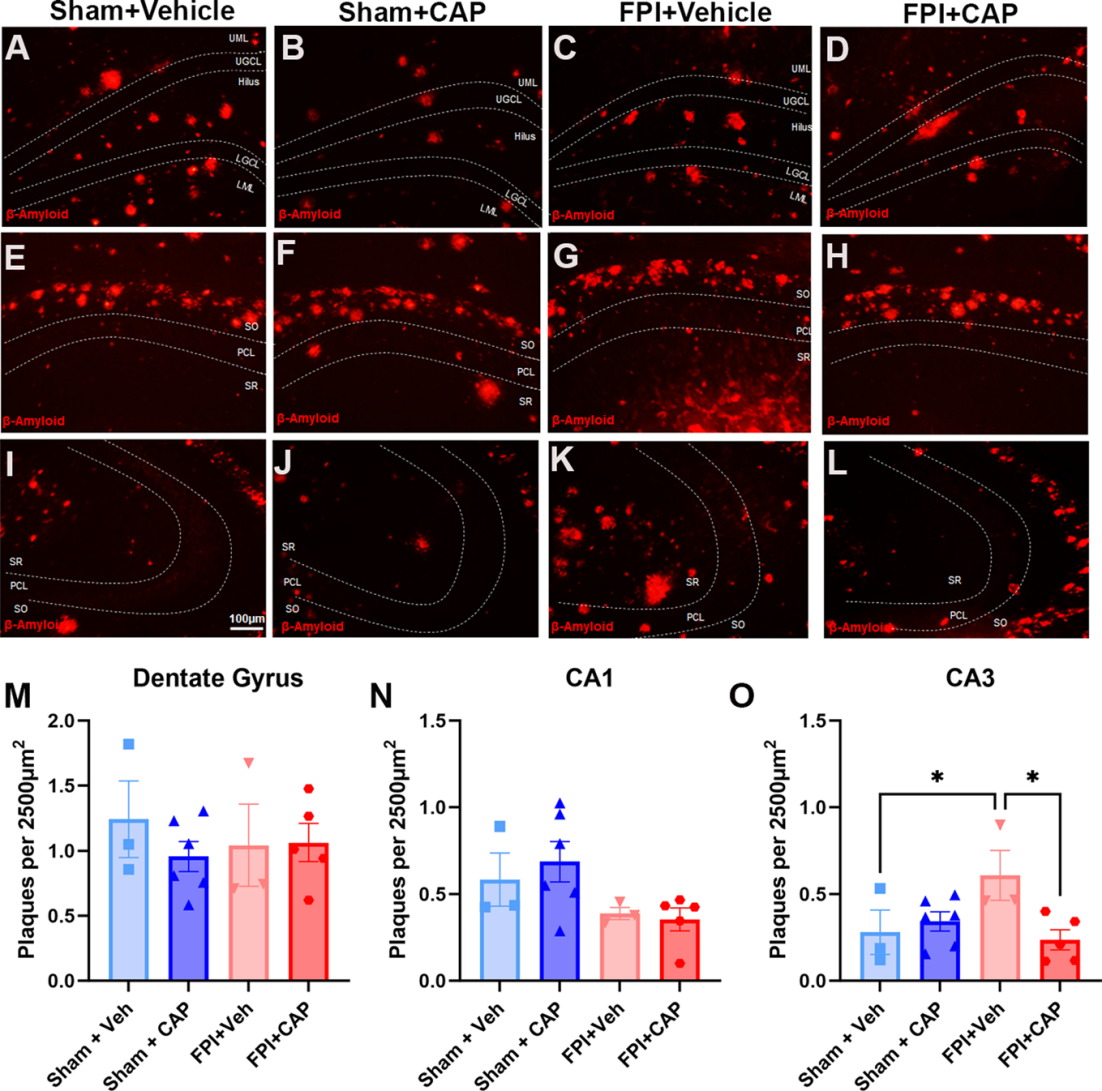
**Hippocampal β amyloid plaques in 5xFAD mice are altered by FPI and CAP in CA3**. Representative micrographs of the DG (**A-D**), CA1 (**E-H**), and CA3 (**I-L**) are shown. There were no significant differences in the DG (**M**), or in CA1 (**N).** Conversely, in CA3 there was a significant increase in 5xFAD mice exposed to FPI (**O**), and CAP significantly prevents this FPI-induced increase in plaques. Scale bar in I = 100 μm for all micrographs. Data are represented as Mean ± SEM; *n* = 3 for vehicle groups, *n* = 6 for CAP groups; *p < 0.05

Amyloid plaques are a pathological hallmark of AD pathology and serve as a marker of AD-associated disease progression [58, 59]. The hippocampus is among the first brain regions to exhibit plaques in clinical AD [60] and the preclinical 5xFAD mouse model [61], the latter of which begin to accumulate hippocampal plaques as early as 2 months of age [62]. In the current study, quantitative analysis of amyloid plaques in the hippocampus confirmed previous reports showing sub-field differences in plaque deposition in 5xFAD mice, including differences in plaque size and number (Supp. Figure 3C). When compared to age-matched 5xFAD Sham + Veh mice, FPI increased the number of plaques in CA3 (*p* < 0.05) (Fig. 4O), while CAP treatment after FPI significantly reduced the number of plaques in CA3 (*p* < 0.05) (Fig. 4O). Other hippocampal subfields, including the dentate gyrus and CA1, did not have FPI-induced changes in total plaque number (Fig. 4M-N) but did differ in size and distribution (Supp Fig. 3).

#### FPI alters microglia in the hippocampus of 5xFAD mice

**Fig. 5:**
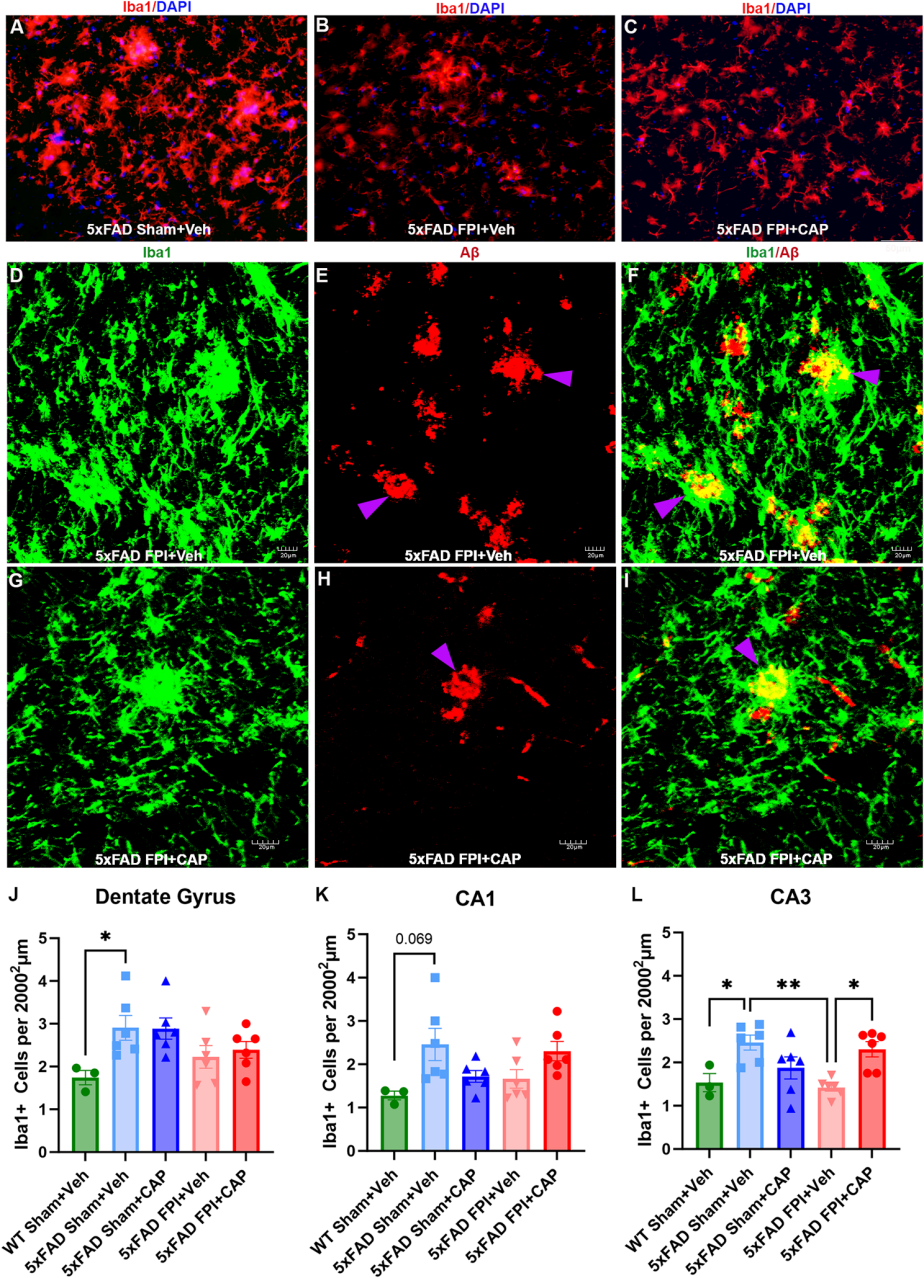
**Increased hippocampal microglial cells in 5xFAD mice are reduced by FPI and restored by CAP in CA3**. In (**A**-**C**), representative micrographs of Iba1 + microglia in CA3. In **D-I**, representative confocal z stacks depict co-staining for Iba1 + microglia and Aβ in 5xFAD FPI + Veh (**D-F**) and 5xFAD FPI + CAP (**G-I**). Note, some of the plaques appear to have been phagocytosed by the microglial cells, as evidenced by plaques within the microglial cytoplasm (purple arrowheads). Quantification of Iba1 + cells found a significant increase of microglial cells in the DG (**J**) and a trend in CA1 (**K**) of 5xFAD mice that is not impacted by CAP or FPI. In **L**, the significant elevation of microglial cells in CA3 is reduced by FPI and restored by CAP treatment after FPI. Scale bar in C = 50 μm for A-C, scale bars in D-I = 20 μm. Data are represented as Mean ± SEM; *n* = 3 for WT Sham + Veh, *n* = 6 for all 5xFAD groups; *p < 0.05, **p < 0.01

Microglia are key mediators of neuroinflammation in both TBI [63–65] and AD [11, 66, 67], and microglia can interact with peripheral immune cells, including B and T cells [68–71]. Consistent with previous data in 5xFAD mice, we observed widespread increases in microglial number throughout the hippocampus compared to WT mice (Fig. 5). There were significant increases in the number of Iba1 + cells found in the DG (*p* < 0.05), CA3 (*p* < 0.05), and a trend in CA1 (*p* = 0.069) (Fig. 5J-L). While there were no effects of CAP or FPI in the DG or CA1, in CA3 there was both a CAP and an FPI effect (Fig. 5L) whereby FPI significantly reduced the number of Iba1 + cells in CA3 (*p* < 0.01), and this decrease was significantly increased by CAP treatment (*p* < 0.05 vs. 5xFAD FPI + Veh) (Fig. 5L). Thus, FPI reduced microglial cells in CA3 and increased plaques in CA3, suggesting that FPI 6 months prior may have altered the ability of CA3 microglial cells to phagocytose plaques. In support of this notion, CAP treatment after FPI increased the microglial cells in CA3 and reduced the plaques. That CAP treatment significantly reduced CD74 + cells in the hippocampus, and CLIP + B cells, but not CLIP + macrophages in the meningeal lymphatic, suggests a relationship between CD74/CLIP, microglial cells, and plaque deposition.

#### Reduced adult hippocampal neurogenesis in the 5xFAD mouse is improved by CAP and exacerbated by FPI

**Fig. 6:**
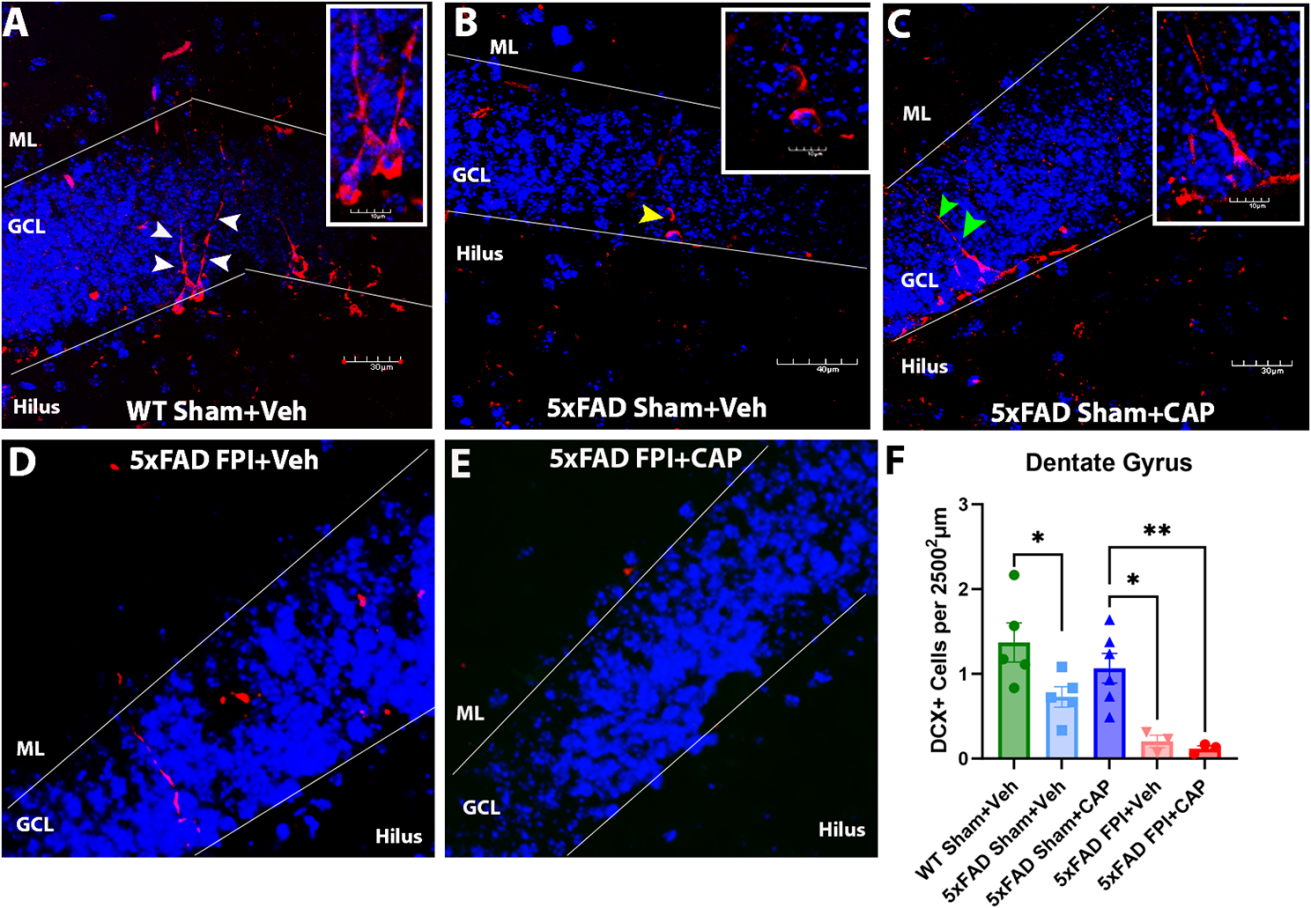
**Reduced hippocampal neurogenesis in 5xFAD mice is improved by CAP and exacerbated by FPI**. In (**A**-**E)**, confocal micrographs illustrating DCX + immature neurons in the dentate gyrus of WT Sham + Veh (**A**), 5xFAD Sham + Veh (**B**), 5xFAD Sham + CAP (**C**), 5xFAD FPI + Veh (**D**), and 5xFAD FPI + CAP (**E**). In **F**, quantitative analysis shows a significant reduction in DCX + cells in the 5xFAD mice that is rescued by CAP. These results also show that FPI exacerbates the reduced hippocampal neurogenesis in 5xFAD mice, and CAP is unable to rescue this exacerbation. Qualitative analysis also reveals alterations in dendrites and their branching through the granule cell layer into the molecular layer in 5xFAD mice (yellow arrowhead) compared to WT (white arrowheads), and this change is partially restored with CAP treatment (green arrowheads). These areas of the images are enlarged in the insets. Data in F are represented as Mean ± SEM; *n* = 3–6; *p < 0.05, **p < 0.01

Reduced adult hippocampal neurogenesis has been previously reported in 5xFAD mice at 2 [72], 4 [73, 74], and 8 [75] months of age. Consistent with these data, quantitative analysis revealed significantly less DCX-labeled immature granule cells in the DG of the 5xFAD Sham + Veh mice (*p* < 0.05) compared to age-matched WT Sham + Veh mice (Fig. 6F). CAP ameliorated the deficit in neurogenesis in 5xFAD sham mice but was unable to rescue the reduction in 5xFAD mice that received an FPI (Fig. 6F). These data mirror the effects of CAP and FPI on the meningeal CLIP + B cells, as seen in Fig. 1.

#### CAP improves chronic FPI-induced cerebrovascular alterations in 5xFAD mice

**Fig. 7:**
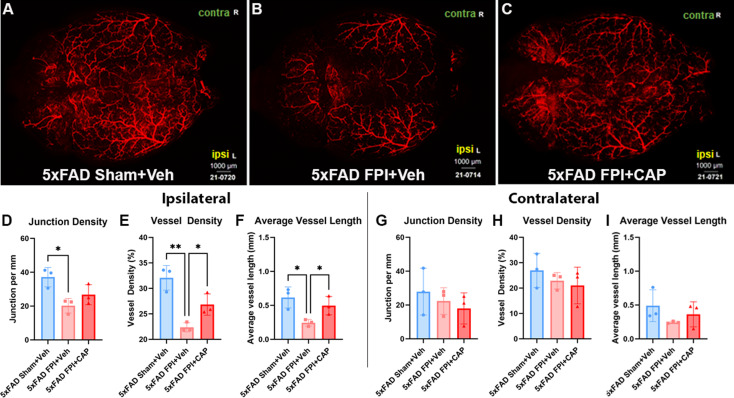
**FPI induces chronic alterations in cerebrovascular structure that are improved by CAP treatment**. In (**A**-**C**), confocal micrographs of vessel painting in 5xFAD Sham + Veh (**A**), 5xFAD FPI + Veh (**B**), and 5xFAD FPI + CAP (**C**). Quantification showed that FPI significantly reduced junction density, vessel density, and average vessel length in the ipsilateral hemisphere (**D-F**). Vessel density and average vessel length were significantly increased in FPI + CAP mice (**E, F**). There were no significant changes identified in the contralateral hemisphere (**G-I**). Scale bars = 1000 μm. Data are represented as Mean ± SEM; *n* = 3 for all groups; *p < 0.05, **p < 0.01

The cerebrovasculature has been found to be altered in clinical and preclinical AD [76–79]. Previous studies have identified chronic vascular alterations in 5xFAD mice compared to age-matched WT, including reduced vessel length and junction density [80]. The results from the current study show significantly altered cerebrovasculature after FPI in 5xFAD mice (Fig. 7). In the ipsilateral hemisphere after FPI in 5xFAD mice, there were significant reductions in junction density (*p* < 0.05), vessel density (*p* < 0.01), and average vessel length (*p* < 0.05), as compared to 5xFAD Sham + Veh (Fig. 7D-F). CAP treatment after FPI significantly increased vessel density (*p* < 0.05) and average vessel length (*p* < 0.05) compared to vehicle treated mice (Fig. 7D-F). No significant changes were noted in the contralateral hemisphere (Fig. 7G-I). Together, these data indicate that FPI chronically exacerbated cerebrovascular alterations in the 5xFAD mouse, and that CAP treatment alleviated these effects.

#### CAP treatment restores altered brain connectome after FPI in 5xFAD mice

**Fig. 8:**
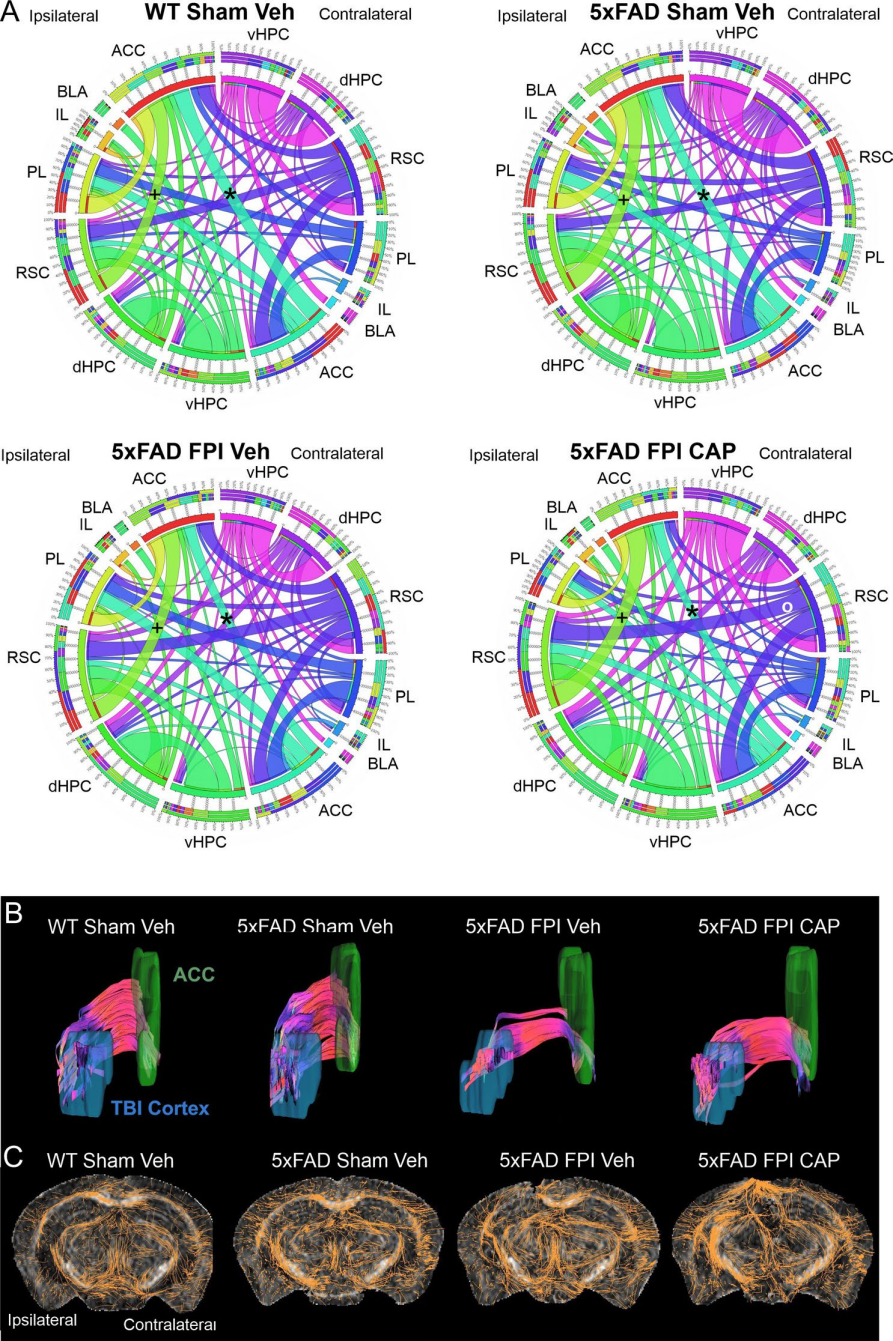
**Altered connectivity in the 5xFAD mouse is affected by FPI and CAP treatment**. In (**A**), connectogram analysis revealed genotype differences between WT Sham + Veh and 5xFAD Sham + Veh mice, including reduced interhemispheric connections between the ACC (*). FPI in 5xFAD + Veh mice showed exacerbated connectivity. In 5xFAD FPI + CAP mice there was a further reduction in bilateral ACC connectivity but increased (presumably compensatory) connections bilaterally in RSC. In (**B**), tract shape analysis revealed that 5xFAD mice have greater tract curl than their WT counterparts. In 5xFAD FPI mice, CAP treatment significantly increased tract density relative to 5xFAD FPI + Veh- mice. In (**C**), 2D reconstructions of tracts illustrate the increased number of tracts in 5xFAD FPI + CAP mice compared to the paucity of tracts in the 5xFAD FPI + Veh mice. Note the increased tract plasticity in the peri-injury area. ACC – anterior cingulate cortex, BLA – basolateral amygdala, dHPC – dorsal hippocampus, IL – Infralimbic prefrontal cortex, PL – prelimbic prefrontal cortex, RSC – retrosplenial cortex, vHPC – ventral hippocampus

To further assess brain alterations in 5xFAD mice following FPI, we investigated cortical connectivity using MRI (Fig. 8). There were clear genotype-related effects on cortical connectivity, such that sham 5xFAD mice exhibited reduced interhemispheric connectivity of the anterior cingulate cortex (ACC), compared to WT sham mice (Fig. 8A). There was also an increased ipsilateral strength of anterior to posterior connections between the ACC and retrosplenial cortex (RSC) (Fig. 8A) in the 5xFAD Sham + Veh mice. This latter effect was also observed in 5xFAD FPI + Veh mice (Fig. 8A). FPI + CAP treated mice exhibited greater interhemispheric connectivity to the RSC than the ACC (Fig. 8A), suggesting that CAP treatment enhances connectivity but not uniformly.

To better understand tracts entering the ACC, we generated tractography reconstructions by seeding the FPI site above the dorsal hippocampus (Fig. 8B). Tract shape analysis revealed that 5xFAD mice had greater curl (total length/Euclidean distance) in their tracts than their WT counterparts (*p* < 0.05, Fig. 8B). FPI in 5xFAD Veh-treated mice resulted in a trending increase in mean diffusivity (MD) (*p* = 0.07, Fig. 8B), with a concomitant increase in radial diffusivity (RD) (*p* = 0.05, Fig. 8B). Treating 5xFAD mice with CAP after FPI significantly increased the number of streamlines relative to WT Sham + Veh controls (*p* < 0.01, Fig. 8B). Overall, there was altered connectivity from the FPI cortical site to the ACC in all FPI groups, as compared to WT Sham + Veh. The 5xFAD Sham + Veh and the 5xFAD FPI + CAP groups exhibited changes in shape analysis metrics [81], while 5xFAD FPI + Veh mice had altered diffusivity metrics. These findings suggest that ACC connectivity in both the 5xFAD Sham + Veh and 5xFAD FPI + CAP groups was altered along the tracts (modified connectivity), while the 5xFAD FPI + Veh mice had microstructural perturbations.

Additional assessment of tract alterations after FPI and CAP treatment were visualized from 2D reconstructions of coronal sections at the injury site (Fig. 8C). These images illustrate the dynamic reorganization of the tracts (shown in orange) after injury on the ipsilateral hemisphere. In this case, this reorganization is more pronounced in the 5xFAD FPI + CAP treated mice than in the 5xFAD FPI + Veh mice. These findings suggest that CAP treatment after injury in 5xFAD mice partially restores the 5xFAD phenotype.

### Behavioral assessments

#### CAP treatment improves acute gait deficits after FPI in 5xFAD mice

**Fig. 9:**
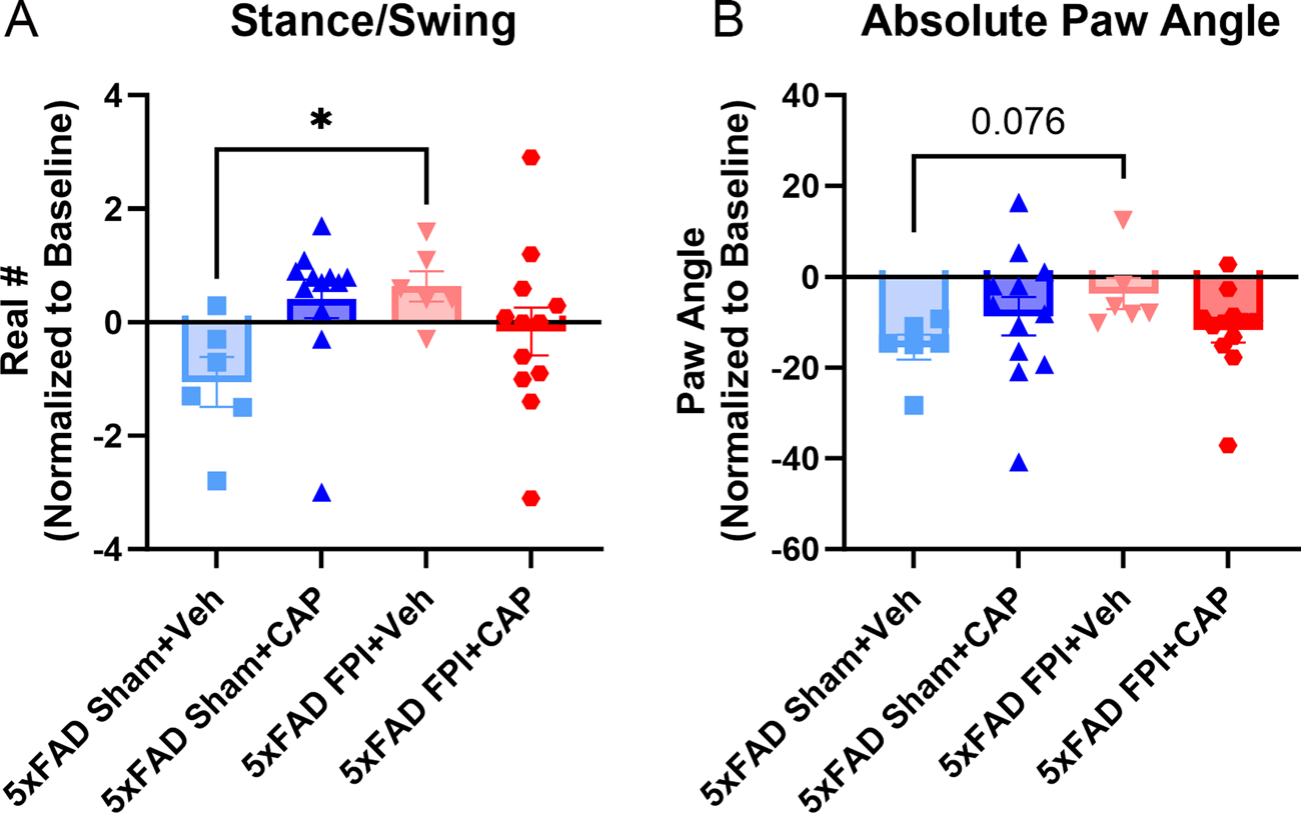
**CAP treatment mitigates FPI-induced acute motor deficits in 5xFAD mice**. Mouse gait was analyzed using the digigait 1 day post injury to assess motor deficits. 5xFAD FPI + Veh mice had significantly increased stance/swing (**A**) and a trending increase in paw angle (**B)** in the right hind leg, indicating an overall change in motor function after injury. These deficits were not apparent in the 5xFAD FPI + CAP mice. Data are represented as Mean ± SEM; *n* = 6 for vehicle groups, *n* = 12 for CAP groups; *p < 0.05

Digigait testing was used to assess acute motor deficits. Consistent with previous observations using this model of FPI [82], 5xFAD FPI + Veh mice exhibited significantly increased stance/swing on day 1 after injury (*p* < 0.05) compared to 5xFAD Sham + Veh (Fig. 9A). This effect was mitigated in the 5xFAD FPI + CAP group (Fig. 9A). 5xFAD FPI + Veh also showed a trending increase in absolute paw angle (*p* = 0.076), showing that the right hind paw was turned outwards (Fig. 9B). The observed preservation of motor function by CAP treatment after FPI is consistent with our previous report showing that CAP treatment reduces the hindlimb-associated cortical lesion size after FPI in WT mice [14].

#### 5xFAD mice exhibit altered affect that is partially improved by CAP treatment, and aggravated by FPI

**Fig. 10:**
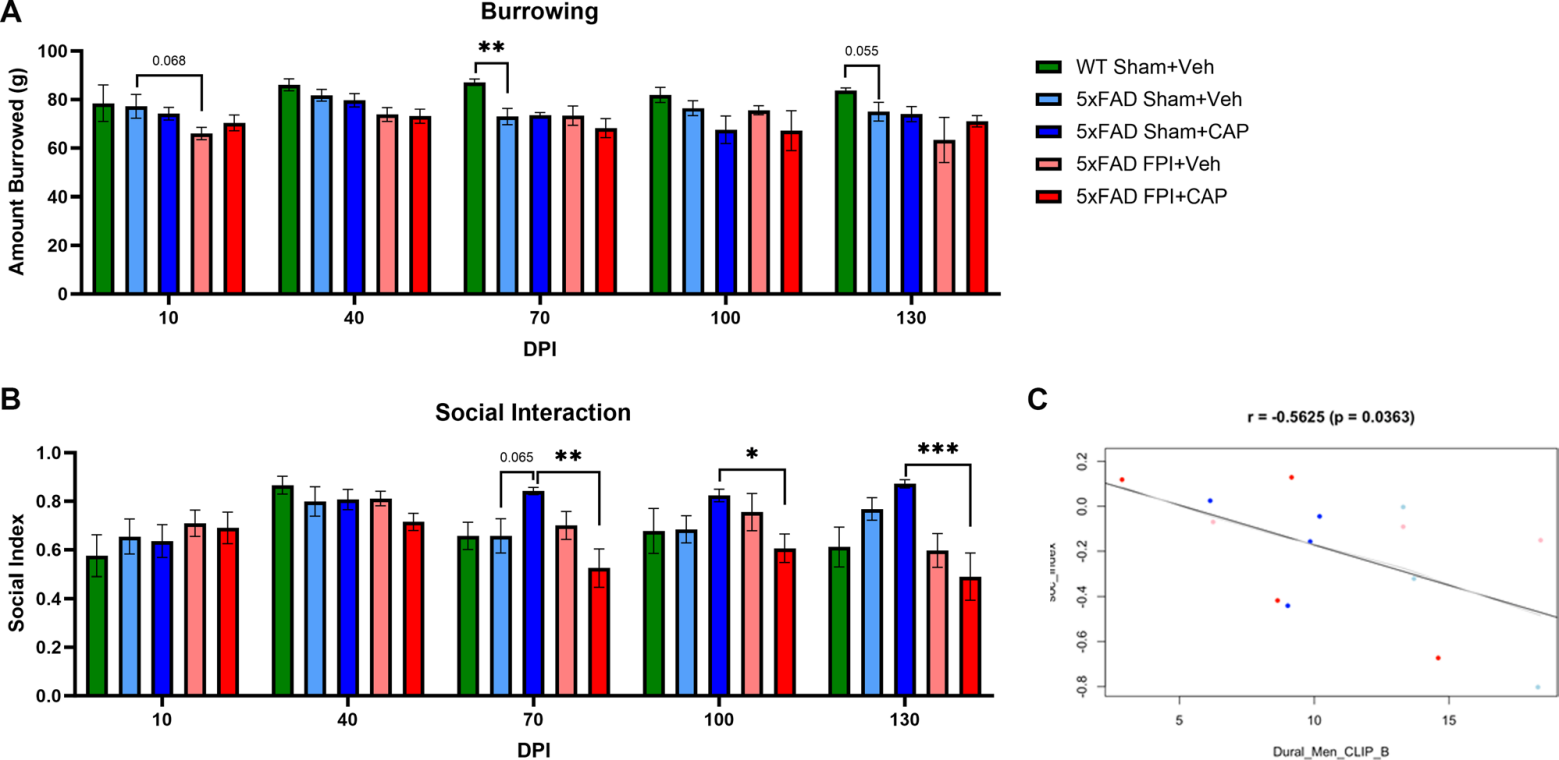
**Differential effects of CAP on affect-associated behavior.** In (**A**), 5xFAD mice exhibit reduced burrowing behavior compared to WT mice, including a significant reduction at 70 DPI and a trend at 130 DPI. Burrowing was reduced after FPI in 5xFAD mice at 10 DPI, but this was not altered by CAP treatment. In (**B**), CAP treatment differentially affects social interaction in 5xFAD mice. CAP increased social interaction in 5xFAD sham mice, most notably at 70 DPI, but reduces social interaction in 5xFAD FPI mice such that 5xFAD FPI + CAP mice exhibited significantly reduced interaction compared to 5xFAD Sham + CAP mice at 70, 100, and 130 DPI. In (**C**), regression analysis revealed a significant negative correlation between social interaction and meningeal CLIP + B cells. Data are represented as Mean ± SEM; *n* = 6 for vehicle groups, *n* = 12 for CAP groups; *p < 0.05, **p < 0.01, ***p < 0.001

More than half of individuals who experience a TBI will develop depression, and depression may be a prodrome of AD [39, 83–85]. We investigated social interaction and burrowing activity beginning at 11 weeks of age and repeated monthly for up to 130 days after FPI and CAP treatment (Fig. 10). 5xFAD Sham + Veh mice exhibited a chronic deficit in burrowing compared to WT Sham + Veh mice (*p* < 0.01) (Fig. 10A; Supp. Figure 1A). This deficit was not improved in 5xFAD Sham + CAP mice. In the 5xFAD FPI + Veh mice, there was a trend towards a reduction in burrowing (*p* = 0.068) compared to 5xFAD Sham + Veh at 10 DPI (Fig. 10A).

Social avoidance was assessed longitudinally using the social interaction test [86, 87]. The results revealed a significant effect of both CAP treatment (*p* < 0.001) and injury (*p* < 0.001) in 5xFAD mice over the longitudinal course of the social interaction assessments. 5xFAD Sham + CAP had a significant increase in social activity over time (*p* < 0.05; Fig. 10B, Supp. Figure 1B), whereas CAP treatment after FPI decreased social interaction in 5xFAD FPI mice (Fig. 10B, Supp. Figure 1B). Exploratory analysis was performed to investigate associations between neurobehavioral testing and pathological endpoints (See Supp. Figure 2 for full exploratory analyses). A significant negative correlation was identified between meningeal CLIP + B cells and the social index (*r*=-0.5625; *p* = 0.0363), indicating increased CLIP + B cells were associated with reduced social interaction (Fig. 10C).

#### Cognitive impairment in 5xFAD mice can be altered by both CAP and FPI

**Fig. 11:**
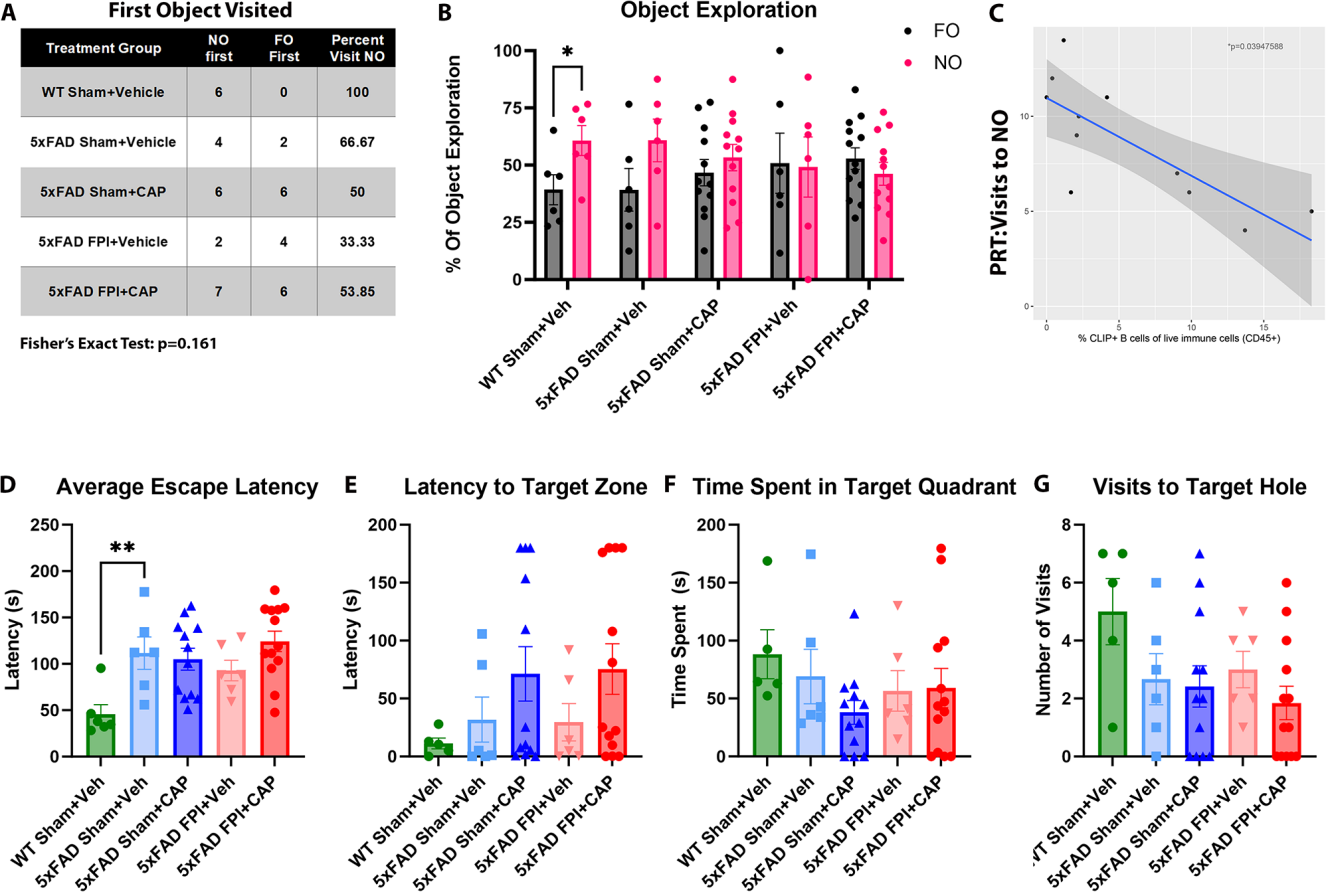
**5xFAD mice exhibit hippocampal-associated cognitive deficits**. In (**A**-**C**), mice were challenged with the pattern recognition test (PRT). The results showed that a reduced percentage of 5xFAD Sham + Veh mice visit the NO first (**A**), and this effect was exacerbated by FPI. In (**B**), WT mice showed a significant preference for the NO that was not found in any of the 5xFAD groups. In (**C**), visits to the NO in PRT exhibited a significant negative correlation with the percentage of meningeal CLIP + B cells. In (**D**), 5xFAD mice had a longer average escape latency in the Barnes maze during the 4 training days, but this is not changed by FPI or CAP treatment. No significant differences were identified in the probe trials (**E-G**). It is pertinent to note that several mice did not visit the target quadrant for the full 3 min. Data are represented as Mean ± SEM; *n* = 6 for vehicle groups, *n* = 12 for CAP groups; *p < 0.05, **p < 0.01

At 8 months of age, 5 months after FPI, mice underwent cognitive behavioral testing in the pattern separation test (PST) and the Barnes Maze to assess hippocampal associated learning and memory. In the PST, 5xFAD Sham + Veh mice exhibit a reduced tendency to visit the novel object (NO) when compared to WT Sham + Veh (66.67% vs. 100%, respectively), and WT Sham + Veh mice exhibited a significant preference for the NO over the FO (*p* < 0.05) (Fig. 11A-B). Neither the 5xFAD Sham + Veh mice, nor the 5xFAD FPI + Veh mice showed any NO preference (*p* = 0.132, *p* = 0.9326, respectively), indicating a failure to recognize the novel object. Further analysis revealed that CLIP + B cells in the dural meninges had a significant negative correlation with the number of visits made to the novel object in the PST (*r*=-0.409; *p* = 0.0395; Fig. 11C), indicating that CAP depletion of meningeal CLIP + B cells is associated with improved PST performance in 5xFAD Sham mice, and suggesting that CLIP + B cells may be a critical population in AD pathogenesis and cognitive impairment.

Consistent with previous studies [88], 5xFAD Sham + Veh mice exhibited impairment in the Barnes maze test (Fig. 11D). This was notable during the first four-days of training where there was a significant increase in average escape latency compared to WT Sham + Veh (*p* < 0.01) (Fig. 11D). This impairment was not improved by CAP treatment or altered by FPI. There were no significant effects identified in CAP or FPI mice on performance in probe trials. These findings suggest a possible floor effect in the 5xFAD mice at this advanced stage of disease progression.

## Discussion

In this study, the pathological and behavioral effects of CLIP antagonism in 5xFAD mice were examined at 6-months after either sham or FPI. The first notable finding is that in the spleen and meninges, CLIP + cells, including CLIP + B cells, are elevated in 9 month-old 5xFAD mice, when compared to age-matched WT mice. A one-time treatment with CAP at 3 months of age significantly depleted the meningeal CLIP + B cell population, but not macrophages, CLIP + macrophages, or splenic CLIP + B cells. Interestingly, the extent of meningeal CLIP + B cells was correlated with decreased social interactions and impaired performance in the PST.

FPI in 5xFAD mice did not chronically alter the B cells, macrophages, dendritic cells, CD4 + or CD8 + T cells in the spleen or meninges. However, it did increase the naïve and central memory CD4 + T cells relative to 5xFAD Sham + Veh mice. These increases may reflect TBI-driven activation and expansion of naïve CD4 + T cells, or TBI-driven reactivation of memory CD4 + T cells. A similar increase in naïve and central memory CD4 + T cells was seen in CAP treated 5xFAD mice. In parallel, CAP treatment seemed to improve some aspects of behavioral performance, including social interaction. It is important to note that the 5xFAD mice exhibit a robust behavioral impairment, particularly in cognitive behavioral testing, and the lack of chronic exacerbation by FPI may be a result of floor effects in some aspects of neurobehavioral performance. It is also possible that, as has been previously reported in FPI models, some aspects of neurobehavioral impairment may be transient. That CAP improved some neurobehavioral outcomes in sham 5xFAD mice suggests that stimulating the transition from an innate to an adaptive inflammatory response early in the pathological progression of 5xFAD mice may ameliorate the adverse effects of chronic inflammation and modify the pathological progression.

While the precise mechanisms of the current findings remain elusive, the observation of altered memory and naïve CD4 + T cells is highly suggestive of the possibility that both CAP and FPI promote antigen processing and presentation by professional antigen presenting cells to T cells, leading to an antigen-specific adaptive immune response. This possibility is supported by the expansion of CLIP + B cells, as well as the trends towards increases in naïve and central memory CD4 + T cells in CAP and FPI mice. That the cumulative effect of CAP and FPI resulted in a significant elevation of these T cell subsets also supports this notion. In the case of CAP, which was designed to outcompete CLIP for the MHCII antigen binding groove [43], it is possible that CAP itself was presented and recognized by T cells, as CAP has been previously shown to promote the transition to an adaptive immune response [43, 44]. However, CAP has also been shown to have anti-inflammatory properties following FPI and these can’t be ruled out as possible therapeutic mechanisms of the present study [14]. Still, CAP inhibited the chronic expansion of some inflammatory immune cell subsets, expanded central memory and naïve T cells, and rescued some of the well-described neuropathology and cognitive deficits in the 5xFAD mice. Future studies are needed to more fully elucidate the immune mechanisms by which CAP exerts beneficial effects.

Another novel discovery in the current study is the identification of CD74 + cells in the hippocampus of 5xFAD mice. The hippocampus is a major component in cognition, learning, and memory, and is a primary area of pathology after TBI, in AD, and in the 5xFAD mouse model [58, 62, 89–96]. CD74, of which CLIP is a cleavage product, has been shown to increase expression on both immune and non-immune cells under conditions of inflammation [50]. However, the role of CD74 + cells that have entered the brain parenchyma has not been previously explored. Therefore, future studies are also needed to address the potential role of CD74 + cells in the hippocampus of 5xFAD mice.

The FPI model, like the vast majority of clinical TBIs, is a closed head injury in which foreign antigens are not introduced into the brain. Therefore, the antigen-specific adaptive immune response reported in preclinical and clinical TBI studies [14, 15, 97] is highly likely to be presenting self-antigens. This is supported by the previous observations of brain specific antibodies in the CSF and serum of TBI patients [98, 17, 99–103]. In the current study, one of the neuropathological changes induced by FPI was the long-term exacerbation of decreased hippocampal neurogenesis observed in the 5xFAD mice. Adult hippocampal neurogenesis has been linked to pattern separation performance [104] and the PST deficit was also exacerbated by FPI. Pattern separation deficits have been previously reported in human AD patients [105–107], and in mouse models of AD [108], including 5xFAD mice. In the latter 5xFAD model, impaired hippocampal neurogenesis has been reported as early as 2 months of age [109]. It is interesting to consider that CA3 pyramidal cells are targets of the dentate granule cell mossy fibers. The accumulation of plaques in CA3 observed in the current study may therefore alter the mossy fiber growth cues leading to reduced generation of newborn granule cells, and this may link the altered adult neurogenesis to the impaired PST performance.

The decrease in microglial cells and increase in plaque deposition after FPI in CA3 of 5xFAD mice is of particular interest and may further implicate CA3 in the hippocampal-related cognitive impairment. CA3 has been linked to Barnes Maze performance [110–113], among other components of spatial learning and memory. One possible interpretation of these findings is that FPI in the 5xFAD mice induced a loss of microglial cells in CA3 that enabled the plaques to increase in size and number. This notion is supported by previous studies that indicate microglial cells can phagocytose plaques and that microglia alter their expression profile in response to plaques [114–116]. Additionally, MHCII + microglia have been implicated in both AD and experimental TBI [117, 118], and the presence of the CD74 + cells also cannot be ruled out as contributing to the loss of Iba1 + cells. Previous studies using the FPI model have also identified pathological alterations to CA3, and further examination of the potential role of CA3 in post-traumatic syndromes is needed.

The cerebrovascular system is a critical nexus through which immune cells interact with the brain [78], and vascular permeability or damage may facilitate the entry of immune cells into the brain parenchyma. Indeed, cerebrovascular damage and dysfunction in both TBI and AD have been linked to immune cell activation and inflammation, including increased migration of peripheral leukocytes into the brain [119, 120]. FPI in clinical and preclinical studies results in acutely altered vascular morphology [121, 122]. Vascular dysfunction is also present early in the pathogenesis of AD, and vascular alterations have been identified in the 5xFAD model [76, 80]. Here, CAP treatment attenuated the chronic FPI-related cerebrovascular changes in 5xFAD mice, highlighting the potential crosstalk between the cerebrovasculature and the activated immune system in the progression of AD-associated pathology after injury.

The observation of alterations in cortical connectivity between 5xFAD mice and age-matched WT mice is consistent with previous clinical and preclinical AD and TBI studies showing altered connectivity between brain regions [123–126]. Novel to our study was the clear genotype-related effect, with 5xFAD exhibiting reduced cortical interhemispheric connectivity, but increased strength of anterior to posterior connections compared to WT. FPI further altered cortical connectivity in 5xFAD mice, and this was modulated by CAP treatment. For example, FPI associated decreases in connectivity between bilateral ACC regions was decreased in 5xFAD mice with CAP, but compensatory increases to the bilateral RSC-RSC connections were increased, as was ipsilateral ACC-RSC connectivity. The ACC is involved in higher-level functions, including reward anticipation, decision making, and emotion, and in cognitive tasks [127]. The ACC connects broadly to multiple regions including the RSC and is also engaged in higher-level cognitive tasks, in particular spatial cognition [128]. That these circuits are altered in 5xFAD mice, and can be further modulated by FPI and CAP, may provide further insight into the deficits identified in neurobehavioral tasks in 5xFAD mice both with and without injury and highlight the importance of immune regulation in these processes.

Taken together, antagonism of CLIP was found to differentially impact AD-associated pathology in 5xFAD mice with and without TBI. In 5xFAD mice without TBI, CLIP antagonism reduced meningeal CLIP + B cells and hippocampal CD74 + cells, enhanced neurogenesis, and improved some aspects of neurobehavioral dysfunction. On the other hand, CLIP antagonism did not affect meningeal CLIP + B cells after FPI in 5xFAD mice or improve most behavioral dysfunction. However, CLIP antagonism after FPI did improve TBI-induced neuropathological changes, including plaques, microglia, cerebrovascular alterations, and altered cortical connectivity. The lack of significant exacerbation by FPI on behavioral impairment may be due to the strong behavioral impairment exhibited in the 5xFAD mice, and/or the transient nature of cognitive impairment after TBI and in preclinical models of TBI. Nevertheless, this study identified meningeal CLIP + B cells as a possible pathogenic mediator in AD pathogenesis, including after TBI. Future studies are needed to more fully investigate the mechanisms responsible and the possible therapeutic implications of targeting this unique immune cell population.

## Materials and methods

### Experimental design

**Fig. 12:**
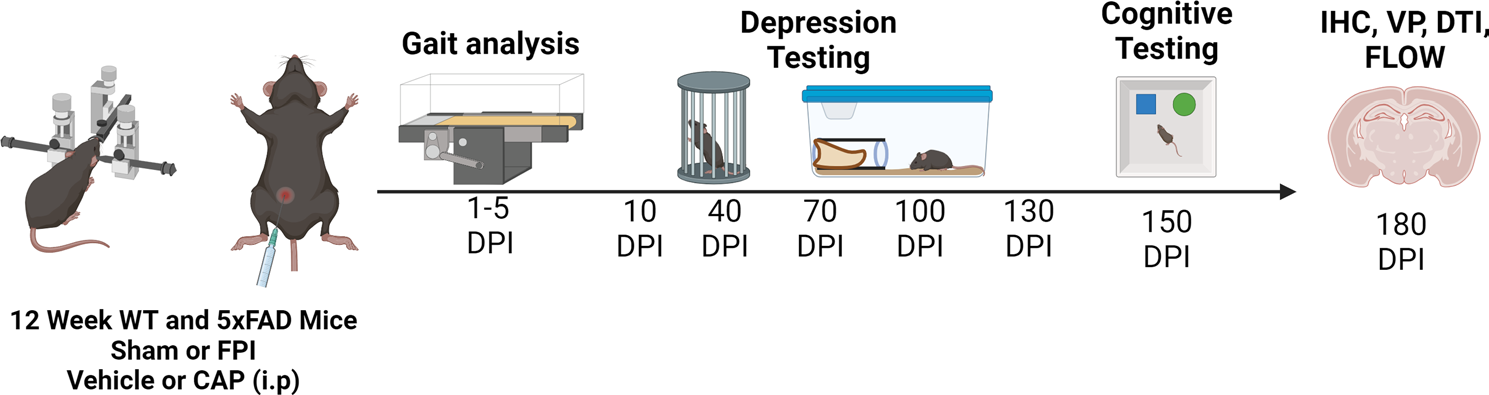
Experimental design. Male 12-week-old wild type (WT) or 5xFAD mice received either fluid percussion injury (FPI) or sham surgery followed 30 min later by treatment with the CLIP antagonist peptide (CAP) or vehicle. Gait analysis was done at 1–5 days post injury (DPI). Depression-associated behavioral testing was done at 10 DPI and every 30 days thereafter. Cognitive testing started at 150 DPI. Tissue collection was done at 180 DPI when the mice were 9 months of age, and tissue was used for a number of pathological analyses including immunohistochemistry (IHC), flow cytometry, vessel painting (VP), and diffusion tensor imaging (DTI)

The experimental design proceeded as depicted in Fig. 12. Following one week of acclimation training, baseline depression testing was performed on all mice at 11 weeks of age. Subsequently, male 5xFAD mice received either lateral FPI as a model of TBI or sham surgery at 12 weeks of age. Thirty minutes after injury, mice were treated once with a single intraperitoneal (i.p.) dose (1 mg/kg) of CAP or Veh. An additional group of age-matched WT male mice received sham injury and vehicle treatment, as a genotype control. For behavioral testing, groups were *n* = 6 for vehicle treatment groups and *n* = 12 for CAP treatment groups. All mice underwent acute monitoring of motor deficits, followed by depression testing 10 days post injury (DPI), and then every 30 days for the next 120 days. At 150 DPI (32 weeks of age), cognitive performance was assessed using the pattern separation test (PST) and Barnes Maze. At 180 DPI (36 weeks of age), the mice were euthanized, and tissue was collected for analysis. A subset of mice (3–4 per group) was used for flow cytometric analysis and others were used for magnetic resonance diffusion tensor imaging (MRI DTI), vessel painting, and immunohistochemistry (3–6 per group).

#### Animals

All work was approved by the Texas A&M Institute for Animal Care and Use Committee (IACUC) under animal use protocol #2020 − 0140. Male Wildtype (WT) C57bl/6J (Jackson Laboratory, Stock #000664) and 5xFAD mice (MMRRC034848 B6.Cg-Tg (APPSwFlLon, PSEN1*M146L*L286V)6799 Vas/Mm) (male, age 9 weeks) were purchased from the Mutant Mouse Resource and Research Center (MMRRC) at Jackson Laboratory and allowed to acclimate for 1 week prior to the experimental start. All mice were housed individually in ventilated cages in a controlled environment and maintained on a standard diet for the duration of the experiment.

#### Fluid percussion injury (FPI)

Lateral FPI was used as a model of TBI and was performed as previously described [14, 30, 129, 130]. Briefly, a 2 mm craniotomy, performed using a stereotaxic device (Stoelting, Wood Dale, IL) under anesthesia, was performed over the left parietal cortex, keeping dura intact. The female end of a luer-lock syringe was cemented over the craniotomy and attached to the FPI apparatus (Custom Fabrication & Design, Richmond, VA). A 12–16 ms FPI was delivered at a pressure of ~ 1.5–1.8 atm. Sham mice received identical treatment, with the exception being that no pressure pulse was delivered. Mice were maintained under isoflurane anesthesia for the duration of the surgical procedure, except during the impact.

#### Drug Treatment

Mice were treated with CAP as previously described [14, 30]. Mice were injected i.p. with 1 mg/kg CAP dissolved in dimethyl sulfoxide (DMSO) and further diluted with sterile saline. Vehicle mice received equivalent volume of DMSO dissolved in sterile saline. All treatments were administered 30 min after the FPI or sham procedure.

#### Digigait

Our FPI model consistently yields a transient contralateral hindlimb deficit that resolves within 5–7 days after injury. To confirm this deficit, the digigait system (Mouse Specifics Inc., Framingham, MA) was used to assess acute changes in gate for the first 5 days after FPI or sham procedure in 5xFAD mice. Mice were acclimated to the room and trained to walk on the digigait at 12 cm/s 3 days prior to surgery. Following FPI or sham, mice walked on the digigait every day for the first 5 DPI, and videos were recorded for analysis. Analysis was done using the digigait software (Mouse Specifics Inc., Framingham, MA), and metrics of interest included paw angle and stride/swing.

### Depression-Associated behavioral testing

The presence of a depression-associated phenotype is often described using multiple tests, since no single test is considered declarative [131–134]. Mice underwent testing for depression-associated behaviors including a social interaction test and burrowing test, modified from previous studies in rats [135, 136]. All mice received 2 days of acclimation for each test at 10 weeks of age, and then one week later underwent baseline testing for all tests. The same depression testing was then repeated at 10, 40, 70, 100, and 130 DPI for all mice.

#### Burrowing

The burrowing test has been used as a measure of fatigue and anhedonia [137], common symptoms of human depression identified by the *Diagnostic and Statistical Manual of Mental Disorders* (DSM) *V* [138], as well as of spontaneous activity/voluntary behavior [135, 139, 140], and has therefore been related to the animal’s overall well-being and a depression-like phenotype in rodents [141]. Burrowing tubes were created using white PVC pipe (2” diameter), all tubes were 5” long. For testing, tubes are filled up to 4” with corncob bedding and then placed in the corner of the animal’s home cage for 15 min. Mice are allowed free access to the burrowing tube for 15 min, after which the tubes were removed. Tubes are weighed before and after testing, and the amount of bedding burrowed out of the tube is calculated. Prior to testing all animals were acclimated once to the empty burrowing tube for 1 h and once to a full burrowing tube for 15 min to ensure interest in the tube and task.1

#### Social interaction test

Social withdrawal is a common element of human depression [142, 143]. In rodents, the social interaction test can be used to assess social avoidance [86, 144]. The social interaction test was performed as previously described with some modifications [87, 145–147]. The test mice were each acclimated to the testing box, a white plexiglass open field box (60 cm x 45 cm), for 5 min. Following acclimation, a social mouse (age matched C57Bl6/J) was added to the testing apparatus within a circular wire cage placed against the wall in the middle of the long side of the open field box. The test mouse was allowed to freely explore the test apparatus for 5 min, and the number of interactions made with the social mouse and time spent interacting with the social mouse were recorded. The corners opposite to the social mouse cage were designated as avoidance zones, and time spent in avoidance and number of visits to avoidance zones were also manually quantified. A social index was calculated as the amount of time spent in social zones minus time spent in avoidance zones divided by the total time spent in social and avoidance zones.

### Neurobehavioral testing

At 32 weeks of age, all mice underwent neurobehavioral testing to assess possible cognitive impairment in tasks related to the hippocampus, the pattern separation test (PST) and Barnes Maze.

#### Pattern separation test (PST)

The PST was used to measure the ability of mice to recognize pattern separation, as previously described [148–150]. The PST was incorporated into this study to assess a hippocampal-dependent task that is linked to intact neurogenesis [104]. The test was comprised of 3 successive trials separated by a 1-hour intertrial interval. Mice were previously acclimated to the open field test box, as described above for the social interaction test. In the first trial, the animal was placed in the open field box with a first set of two identical objects (shape 1 objects) positioned on floor pattern 1 (P1) and was allowed to freely explore both objects for 5 min. In the second trial, mice were placed in the open field box with a second set of identical objects (shape 2 objects) on floor pattern 2 (P2) and again allowed to freely explore both objects for 5 min. In the third and final trial, one of the shape 2 objects from trial 2 was replaced with a shape 1 object on P2. This shape became the novel object (NO) and shape 2 object became the familiar object (FO) on the P2 floor. Again, the mice were allowed to freely explore both objects for 5 min. Each trial was video recorded and analyzed using automated NOLDUS EthoVisionXT video tracking software. Additional scoring was done manually, in which the visits counted to each object were counted. Along with the number of visits to each object, the latency to visit the NO and the first object visited by each mouse was recorded.

#### Barnes Maze

The Barnes maze is used as an assessment of spatial learning and memory in rodents [88, 151, 152]. Briefly, the maze consists of a brightly lit, white elevated circular platform with equally spaced holes along the perimeter. A non-visible escape box is placed under one of the holes. Mice completed a habituation trial to become familiar with the maze and the environment, and practice entering into the escape box. In the learning phase, mice were trained to find the escape hole. Mice completed 2 trials each day, separated by a 15 min intertrial interval, for 4 consecutive days. Each trial was a maximum of 3 min. If successful, the mouse was rewarded with 1 min in the covered escape box in the dark. If not successful, the mouse was guided to the escape box at the end of 3 min and allowed to sit for 1 min in the dark. The number of incorrect holes visited (errors) as well as the latency to find the escape box was recorded for each trial and averaged for each day for each mouse. 72 h after the last training trial, a probe trial in which the escape box was removed was conducted. During the probe trial, mice were allowed to freely explore the maze for 3 min. Each trial was video recorded and analyzed using automated NOLDUS EthoVisionXT video tracking software. Additional scoring was done manually, in which latency to the escape hole and visits to additional holes were counted.

### Immunohistochemistry

**Fig. 13:**
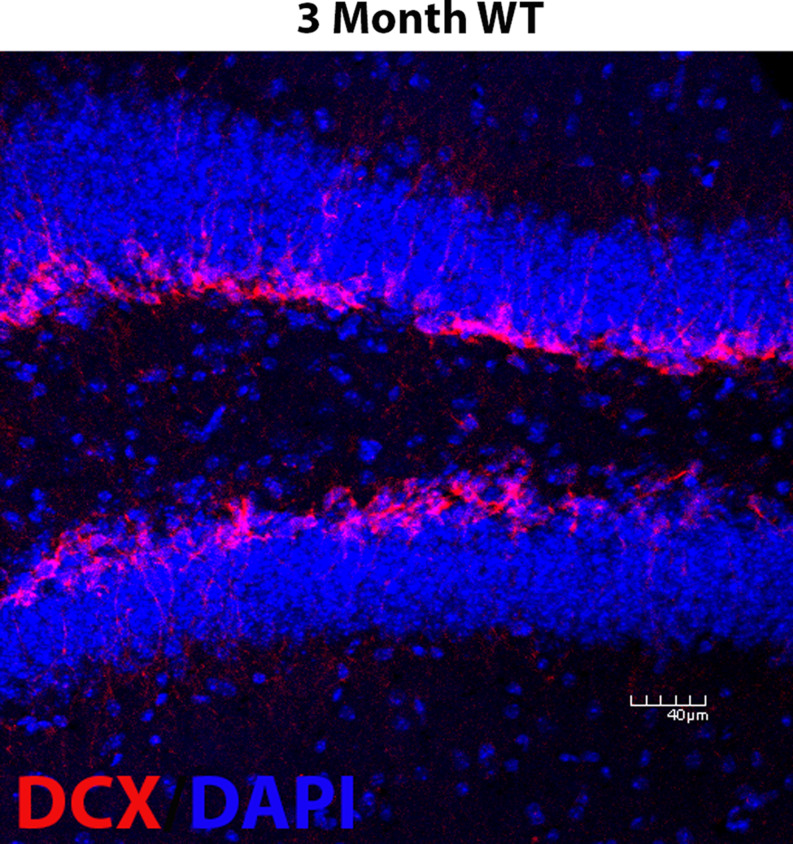
DCX staining in 3 month old WT mouse. To confirm the efficacy of our DCX staining protocol, we stained tissue from a 3 month old WT control mouse and found normal, robust DCX staining in the GCL of the dentate gyrus. This confirms our protocol and highlights the low number of DCX+ cells in our aged mice

Immunohistochemistry for doublecortin (DCX) + immature neurons, Iba1 + microglia, CD74 + cells, and amyloid-β plaques was used, as previously described [148, 153–155]. Briefly, mice were anesthetized with Fatal Plus (Sodium Pentobarbital; 52 mg/kg, administered i.p.) and transcardially perfused with phosphate buffered saline (PBS) through the left ventricle until the blood ran clear. This was followed by 4% paraformaldehyde (PFA) through the left ventricle. All brains were allowed to postfix in the skull for 24 h in PFA, after which they were extracted and fixed for an additional 24 h in 4% PFA. Fixed brains were cut into 44-µm thick serial sections with a freezing microtome (American Optical Corp; Model #860). For DCX and CD74, slices were first incubated in a 1X Citrate Buffer (Millipore Sigma) for 1 h at 45 °C. Slices were then washed and stained with goat anti-DCX antibody (Santa Cruz Inc. USA), rat anti-CD74 (BD Biosciences), rabbit anti-Iba1antibody (Fujifilm Wako Chemicals USA Corp), or rabbit anti-amyloid-β (Cell Signaling Technology) overnight at room temperature, rotating. After overnight incubation, slices were washed and stained with and secondary biotinylated goat anti-Rabbit IgG (Alexaflour-555) (Iba1, amyloid-β), secondary donkey anti-Goat IgG (AlexFlour-555) (DCX), or secondary biotinylated goat anti-Rat IgG (Alexfluor-555) (CD74). Antibodies used are shown in Table 1 Slices were mounted and cover-slipped with antifade reagent containing DAPI (Vector Laboratories #H-1200-10). For reference, we stained 3-month-old WT mice (Fig. 13) simultaneous with all other tissue to confirm the efficacy of our previously described DCX-staining protocol [94, 148, 153, 155].

**Table 1:** Antibodies for immunohistochemistry

Antibody	Species	Dilution	Company (Catalog #)
Iba1	Rabbit	1:400	Wako Labs (019-19471)
Aβ	Rabbit	1:500	Cell Signaling (8243)
Aβ	Mouse	1:500	BioLegend (803,001)
CD74	Rat	1:200	BD Pharmingen (555,317)
DCX (C)	Goat	1:200	Santa Cruz (sc-8066)
DCX (N)	Goat	1:200	Santa Cruz (sc-8067)
Anti-rabbit AlexaFluor-555	Goat	1:200	Invitrogen (A21428)
Anti-rabbit AlexaFluor-488	Goat	1:200	Invitrogen (A11070)
Anti-Rat AlexaFluor-555	Goat	1:200	Invitrogen (A21434)
Anti-Goat AlexaFluor-555	Donkey	1:200	Invitrogen (A21432)

#### Quantitative and semiquantitative analysis

Imaging for all immunohistochemistry was done on a fluorescent microscope (Olympus, Bethlehem, PA). Unbiased stereology-based analysis was used to quantify cells positive for DCX, Iba1, or amyloid-β plaques in the hippocampus, as previously described [30, 94, 148, 153, 156]. Sections (~ every 260–350 μm apart) containing the dorsal hippocampus (Bregma − 1.34 through − 2.80) were selected for analysis. Analysis of DCX + cells was performed in the ipsilateral infra- and supra-pyramidal blades of the DG granule cell layer/subgranular zone, as previously described [94, 148]. Iba1 and amyloid-β analyses were done in CA1, CA3, and the dentate gyrus (DG) hippocampal subregions in the ipsilateral hemisphere. A minimum of 3 left hippocampi were counted per animal, per antibody, within the stereological coordinates indicated above. We also performed semi-quantitative assessment of CD74 + cells in the ipsilateral hippocampus. Slices were scored on a scale of 1–4, and a minimum of 3 slices per animal were evaluated. We further sought to qualify the identification of the CD74 + cells by double-staining with either GFAP for astrocytes or Iba1 for macrophage/microglia.

### Flow cytometry

#### Dural meninges collection and isolation

Mice were perfused with PBS for approximately 5 min, until liver was cleared and the blood ran clear. The skull was rapidly removed and meninges (dura mater, arachnoid, and pia mater) along the superior sagittal sinus were collected using forceps and placed in PBS + 10% Fetal Bovine Serum (FBS) on ice while remaining samples were collected. Following collection, solutions of digestion enzymes containing collagenase 4 and DNAse 1 were prepared. All dura were washed once with PBS, then incubated in digestion enzymes for 1 h at 37 °C. At the conclusion of 1 h, the reaction was quenched by adding the enzyme mix to PBS + 10% FBS. All samples were subsequently spun and resuspended in the desired buffer for flow cytometry.

#### Spleen collection and isolation

Splenocytes were isolated as previously described with minor modifications [14, 30]. Briefly, mice were euthanized, and spleens were removed. Tissues were dissociated, single cell suspensions prepared, and red blood cells were lysed using ACK lysis buffer. The dissociated splenocytes were then counted and stored for subsequent analysis via flow cytometry.

#### Staining of single cell suspensions

Splenic and meningeal cells were analyzed by flow cytometry as previously described [14, 30]. All cells were treated with FC Block (BD Bioscience) prior to staining. Meninges were stained with the following antibodies: CD19 (6D5, BioLegend), CD90.2 (53 − 2.1, BioLegend), CD45 (30-F11, BioLegend), and CLIP (15G4, Santa Cruz Biotechnology). Due to the limited number of cells found in the meninges, only limited immune cell subsets could be examined. More expansive immune cell analysis was performed on splenocytes from these same mice. These included antibodies to the following immune cell subsets: CD19 (6D5, BioLegend), CD90.2 (53 − 2.1, BioLegend), CLIP (15G4, Santa Cruz Biotechnology), CD4 (RM4-5, BioLegend), CD8 (53 − 6.7, BioLegend), CD44 (IM7, BioLegend), CD62L (MEL-14, BioLegend), Ly49 (RA3-6B2, BioLegend), Cd11b (M1/70, BioLegend), Cd11c (N418, BioLegend), and MHCII (M5/114.15.2, BioLegend). Live cells were assessed using Ghost Dye Red 780 viability dye (Tonbo Biosciences). The cells were analyzed using a Becton Dickson Fortessa X-20 flow cytometer and data were analyzed using FlowJo software (TreeStar Inc.) Antibody details for flow cytometry are found in Table 2.

**Table 2:** Antibodies for flow cytometry

Antibody	Clone	Fluorophore	Dilution	Company (Catalog #)
GhostDye (Live/Dead)		Red780	1:100	Tonbo Biosciences (13-0865)
CD19	6D5	PE	1:200	BioLegend (115,507)
CD19	6D5	BV421	1:200	BioLegend (115,538)
CD3	145-2C11	BV421	1:200	BioLegend (100,335)
CD90.2	53-2.1	BV510	1:200	BioLegend (140,319)
CLIP	15G4	FITC	1:100	Santa Cruz (sc-53,946)
MHCII	M5/114.15.2	PerCp-Cy5.5	1:100	BioLegend (107,624)
CD11c	N418	PE	1:100	BioLegend (117,308)
CD11b	M1/70	BV711	1:200	BioLegend (101,241)
CD45	30-F11	BV605	1:300	BioLegend (103,140)
CD4	RM4-5	BV605	1:100	BioLegend (100,547)
CD8α	53-6.7	BV711	1:100	BioLegend (100,747)
CD44	IM7	PE	1:20	BioLegend (103,024)
CD62L	MEL-14	BV421	1:100	BioLegend (104,435)
Ly49 C/F/I/H	14B11	PE/Cy7	1:100	BioLegend (108,209)
CD45R/B220	RA3-6B2	BV785	1:300	BioLegend (103,246)
CD16/CD32 (FC Block)	2.4G2	N/A	1:50	BD Pharmingen (553,142)

### Vessel painting methods and analysis

Analysis of the cerebrovasculature was done using vessel painting methods and analysis as previously described with slight modification [79, 80, 157]. Mice were injected i.p. with heparin and sodium nitroprusside (SNP) and were anesthetized with an i.p. injection of Fatal Plus (Sodium Pentobarbital; 52 mg/kg). Vessel painting was performed by injecting a solution of 1,1′-dioctadecyl-3,3,3′3′-tetramethylindocarbocyanine perchlorate (DiI) (0.3 mg/mL in PBS containing 4% dextrose, total volume of 500 µL) into the left ventricle, followed by a 50 mL PBS flush and a 50 mL 4% PFA perfusion, using a peristaltic pump (8.4 mL/min). The brains were post-fixed in the skull for 24 h, then the brains were extracted, and fixed for an additional 24 h in 4% PFA, washed in PBS, and stored at 4 °C in PBS until imaging. Successfully vessel painted brains were selected if they showed uniform pink staining and excellent staining of large and small vessels on the cortical surface, as previously described [157].

The brains were imaged using a fluorescence microscope (Keyence BZ-X810, Keyence Corp, Osaka, Japan). Axial images of the entire brain were acquired at 2× magnification using the Z-stack feature (~ 42 images, step size 25.2 μm). Classical vessel analysis was performed by using the Angiotool software (Version 0.6a), allowing for measures of vessel density, length, and number of junctions [158], and the ImageJ plugin “FracLac” was used to analyze vascular complexity through fractal analysis [159].

### Magnetic resonance imaging (MRI) via diffusion tensor imaging (DTI)

#### MRI acquisition

Four groups of mouse brains (WT Sham + Veh, 5xFAD Sham + Veh, 5xFAD FPI + Veh, and 5xFAD FPI + CAP; 6 per group) were imaged on a 9.4T Bruker Avance imager (Paravision 5.1, Bruker Biospin, Billercia, MA). Diffusion tensor imaging (DTI) and T2-weighted imaging (T2WI) scans were acquired for 1 h 52 min and 38 min, respectively. Both scans were acquired with the same spatial resolution: 1.5 cm field of view, 0.5 mm slice thickness, and a 128 × 128 acquisition matrix. DTI images were collected with repetition time/echo time (TR/TE) = 8000 msec/35.7 msec, and 30 directions (5 b = 0mT/m, b = 3000mT/m). T2WI scans were acquired with 10 echoes and TR/TE = 4000 msec/10 msec.

#### MRI analysis

Experimenters were blinded to group designations during MRI analysis. The skull was stripped from the scan using ITK-SNAP (version 3.6.0) [160]. DTI images then underwent correction for bias field inhomogeneities [161] and eddy current distortions. Using FMRIB’s Diffusion Toolbox from FMRIB’s Software Library (FSL), diffusion tensor models were assigned to each voxel to generate DTI parametric maps: fractional anisotropy (FA), axial (AxD), mean (MD), radial (RD) diffusivity. In the T2WI scans, bias field inhomogeneities were corrected and quantitative T2 relaxation maps were generated with JIM software (RRID: SCR_009589, Xinapse Systems Ltd; West Bergholt, Essex; United Kingdom). A modified Australian Mouse Brain Mapping Consortium (AMBMC) atlas with 40 bilateral regions (Ullman et al., 2013, Richards et al., 2011) was non-linearly registered to each scan and regional labels were applied with the Advanced Normalization Tools (ANTs). The atlas overlay was manually inspected, and misalignments were corrected with ITK-SNAP. Regional metric outliers within each group were excluded with 1.5xIQR above the first quartile or below the third quartile.

To assess the effects of the various treatments on structural connectivity, DTI scans were reconstructed on DSI studio with a diffusion sampling length ratio of 0.85 (Jan 25, 2022, build; http://dsi-studio.labsolver.org/). Whole brain seeding was performed and manually inspected for tract quality. The AMBMC region designations were then used to generate structural connectivity matrices. Groups were averaged and plotted with Circos (http://circos.ca/ [162]), . Connectivity symmetry was evaluated using an Asymmetry Index ([Left-Right/Left + Right] ×100%). For tractography, DTI scans were reconstructed using a diffusion sampling length ratio of 0.75 for connections between site of injury and anterior cingulate cortex (ACC). The seed was manually delineated in the cortex above the dorsal hippocampus and the target ACC region was generated from the AMBMC atlas. Deterministic tractography was performed using an angular threshold of 70, a step size of 0.06, and 0.80 smoothing. Tract confirmation utilized viral tracing experiments from the Allen Brain Institute (https://connectivity.brain-map.org/) where our tracts closely matched those of experiment #126,907,302.

### Statistical analysis

Power analysis was done using GPower Software (Version 2.0) to determine necessary samples sizes and found that *n* = 12 was sufficient to achieve β = 0.836 in our neurobehavioral assays, also allowing for sufficient sample sizes for subsequent IHC and flow analyses. Due to limitations encountered when running the study, the actual sample size for vehicle-treated groups was *n* = 6 for neurobehavioral assays. While low, this sample size still allowed for effect size d = 0.94 in the Barnes Maze for WT Sham + Veh vs. 5xFAD Sham + Veh. Subsequent statistical analysis was carried out using both GraphPad Prism (Version 9.0) and R. For analysis of WT Sham + Veh versus 5xFAD Sham + Veh, a student’s t-test was used to assess genotype differences. Further analysis of the effects of CAP and FPI on the 5xFAD mice for flow cytometry, behavior, immunohistochemistry, and vessel painting data were analyzed by one-way analysis of variance (ANOVA) with post-hoc testing using the Holm-Sidak correction for planned comparisons. For digigait, the means and standard deviation for each day for each metric were calculated using Python, and a student’s t-test was used to assess differences in pre-planned group comparisons. Generalized estimating equation (GEE) models were used for regression modeling of the burrowing and social interaction longitudinal variables, with natural cubic splines for representing time courses as smooth functions [163]. In the GEE models, interactions were included between the time course curves, surgery (FPI / Sham), and drug (CAP / Vehicle). Tests were conducted for both surgery and drug effects in terms of overall differences between mean time course curves via F-tests of nested models. For PST, Fisher’s Exact Test was used to test for differences in proportions of mice that visited novel objects. Additional exploratory statistical analysis was completed in R to investigate correlations between pathological endpoints and behavioral data via. For all statistical testing, significance was considered *p* < 0.05 and a trend was considered 0.05 < *p* < 0.10.

### Electronic supplementary material

Below is the link to the electronic supplementary material.

Supplementary Material 1

Supplementary Material 2

Supplementary Material 3

Supplementary Material 4

## Data Availability

The datasets generated during and/or analyzed during the current study are available from the corresponding author on reasonable request.

## Abbreviations

ACC: Anterior Cingulate Cortex
AD: Alzheimer’s disease
ANOVA: one-way analysis of variance
AxD: axial diffusivity
CAP: CLIP antagonist peptide
CLIP: Major Histocompatibility Complex class II-associated invariant peptide
DCX: doublecortin
DG: dentate gyrus
DiI: 1,1′-dioctadecyl-3,3,3′3′-tetramethylindocarbocyanine perchlorate
DMSO: dimethyl sulfoxide
DPI: days post injury
DSM: Diagnostic and Statistical Manual of Mental Disorders
DTI: diffusion tensor imaging
EPM: elevated plus maze
FA: fractional anisotropy
FBS: fetal bovine serum
FPI: fluid percussion injury
GEE: generalized estimating equation
i.p.: intraperitoneal
MCI: mild cognitive impairment
MD: mean diffusivity
MHCII: Major Histocompatibility Complex class II
MRI: magnetic resonance imaging
PBS: phosphate buffered saline
PFA: paraformaldehyde
PST: pattern separation test
RD: radial diffusivity
RSC: Retrosplenial Cortex
SNP: sodium nitroprusside
TBI: Traumatic brain injury
WT: Wild type
