# Supplementary figures and images for: Traumatic brain injury alters the effects of class II invariant peptide (CLIP) antagonism on chronic meningeal CLIP + B cells, neuropathology, and neurobehavioral impairment in 5xFAD mice
Source: J Neuroinflammation. 2024 Jun 27;21:165. doi: 10.1186/s12974-024-03146-z (PMC11212436; doi:10.1186/s12974-024-03146-z)

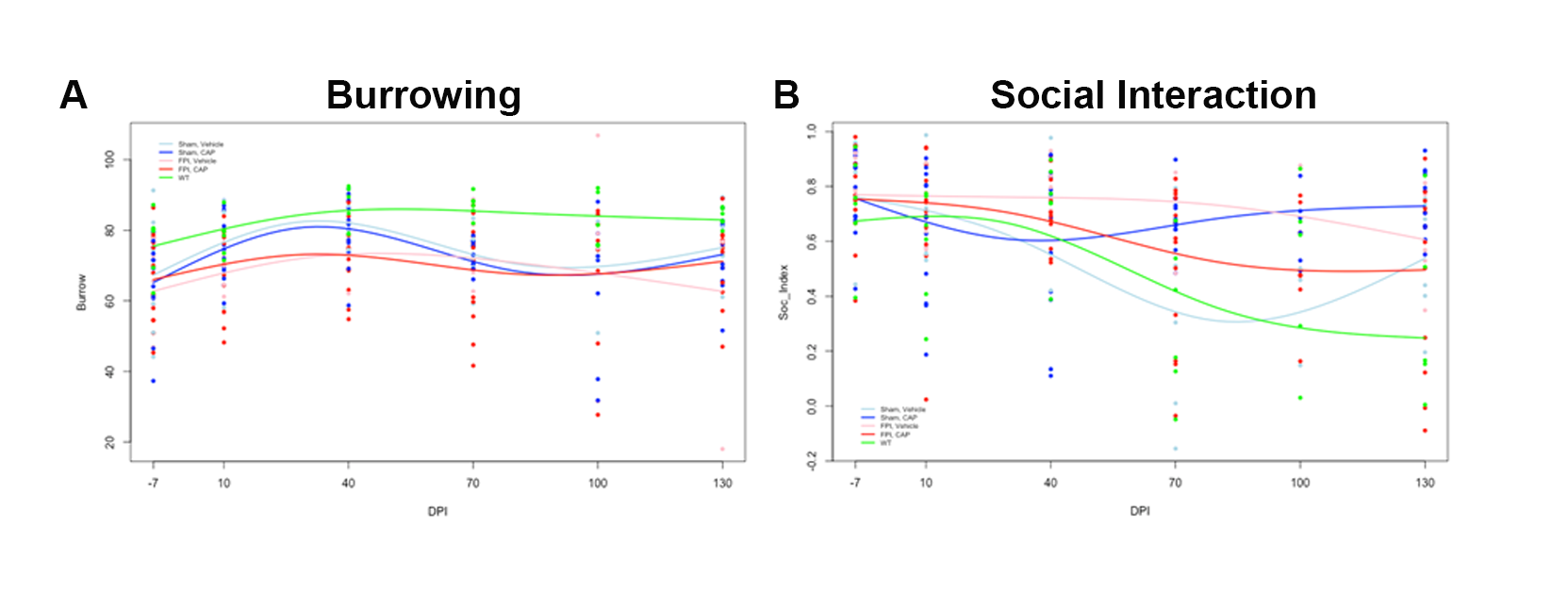

Supplement: Supplementary file 1 — Supplementary Material 1 [file 12974_2024_3146_MOESM1_ESM.tif]

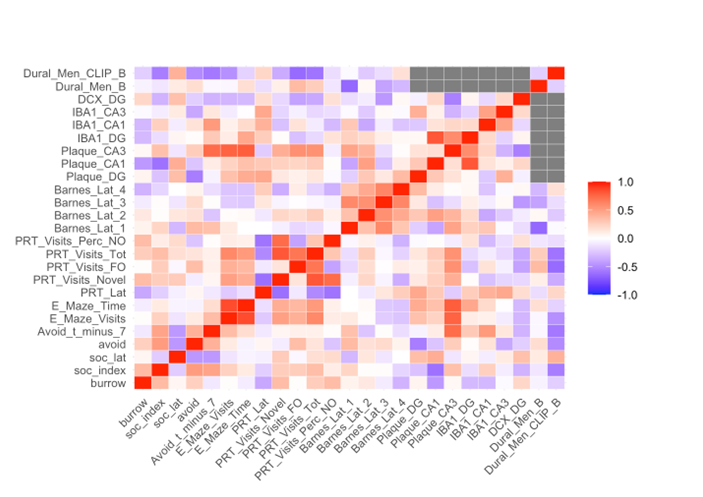

Supplement: Supplementary file 2 — Supplementary Material 2 [file 12974_2024_3146_MOESM2_ESM.tif]

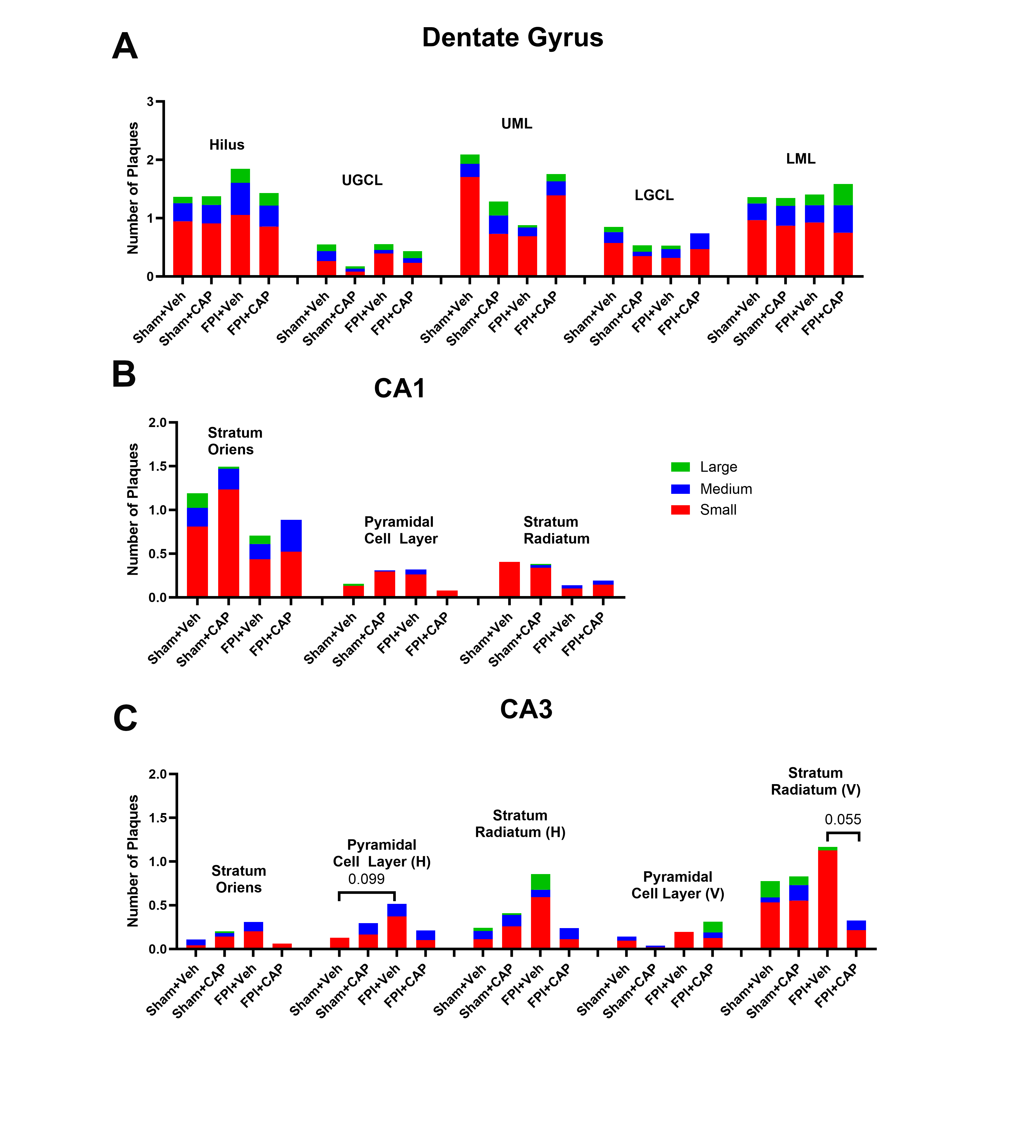

Supplement: Supplementary file 3 — Supplementary Material 3 [file 12974_2024_3146_MOESM3_ESM.tif]
