# Supplementary material for: Traumatic brain injury alters the effects of class II invariant peptide (CLIP) antagonism on chronic meningeal CLIP + B cells, neuropathology, and neurobehavioral impairment in 5xFAD mice
Source: J Neuroinflammation. 2024 Jun 27;21:165. doi: 10.1186/s12974-024-03146-z (PMC11212436; doi:10.1186/s12974-024-03146-z)
Supplement: Supplementary file 4 — Supplementary Material 4 [file 12974_2024_3146_MOESM4_ESM.docx]

**Supplementary Figures**


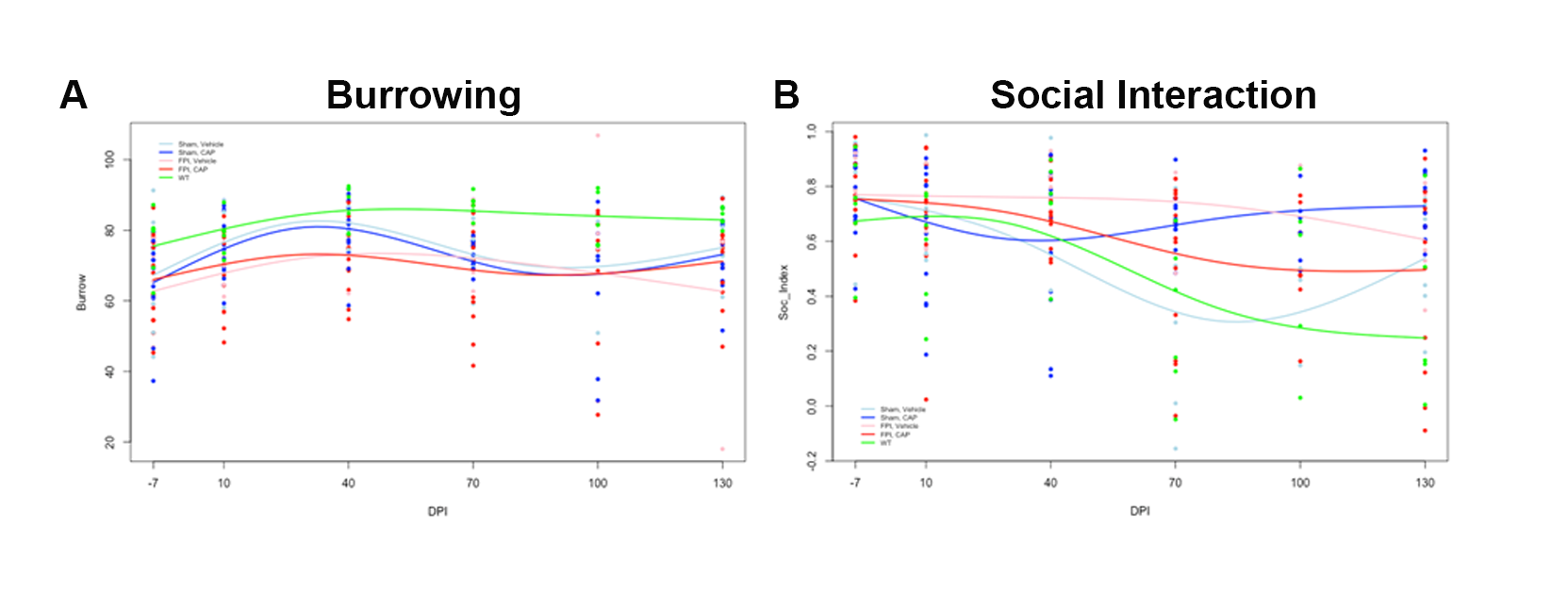


**Supplemental Figure 1. Longitudinal analysis of depression-associated behaviors.** In addition to differences at individual timepoints, longitudinal analysis was done to determine differences in behavior over time for burrowing and social interaction. In **A,** 5xFAD mice exhibited chronic reduced burrowing compared to WT, but there were no effects of CAP or injury. In **B,** there were significant effects of both CAP and FPI on social interaction, such that CAP increased social interaction longitudinally in sham mice, but CAP reduced social interaction longitudinally in FPI mice.


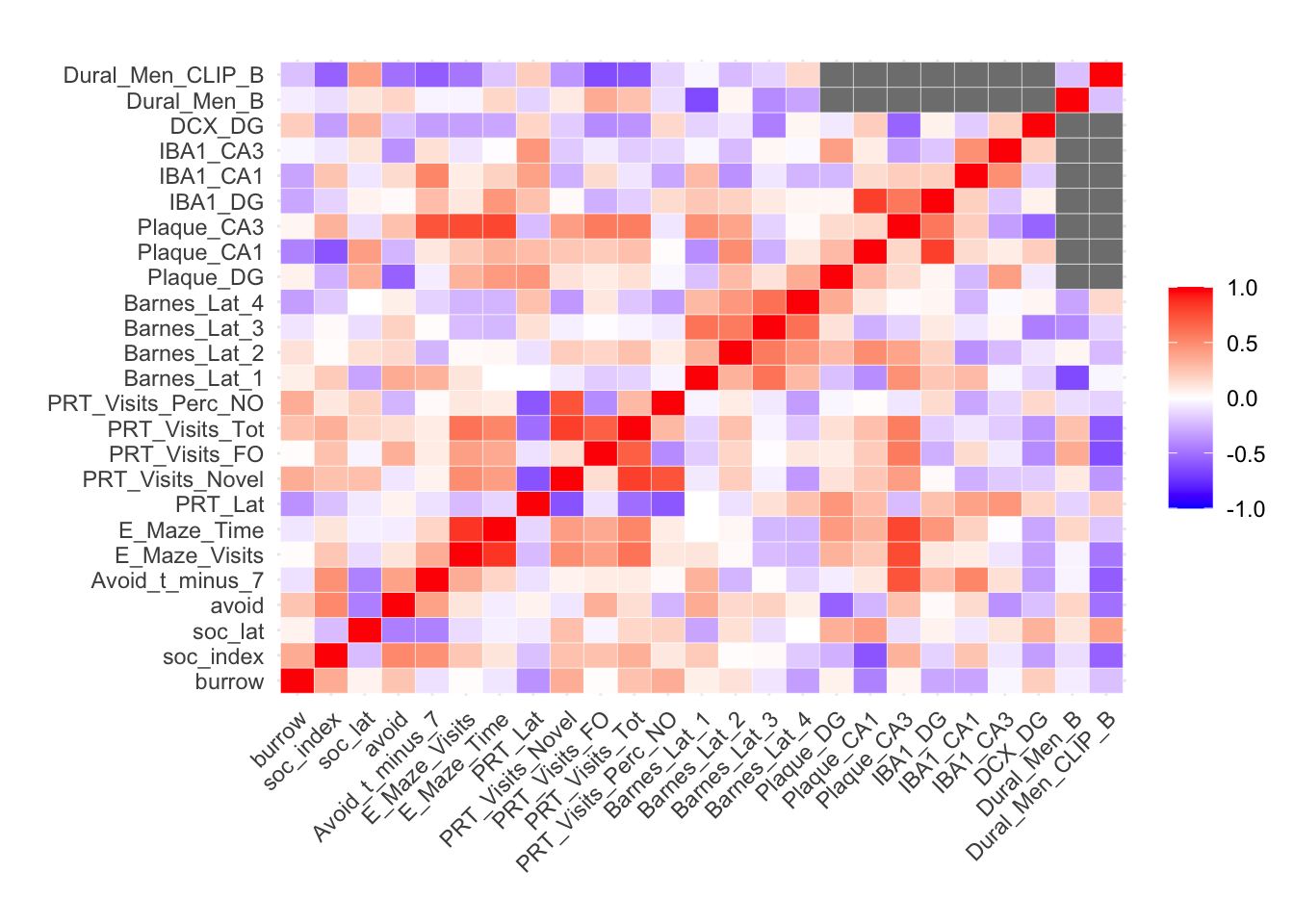


**Supplemental Figure 2. Pearson correlation analysis for behavior- and neuropathological- variables.** As part of our exploratory analysis, a series of analyses were performed. These results are shown in a heatmap of Pearson correlations (r) between all numeric variables (the same as those used in the cluster analysis). Greyed cells correspond to pairs of variables for which there was insufficient data. For example, the animals that were used for flow cytometric analysis were not used for immunohistochemistry.


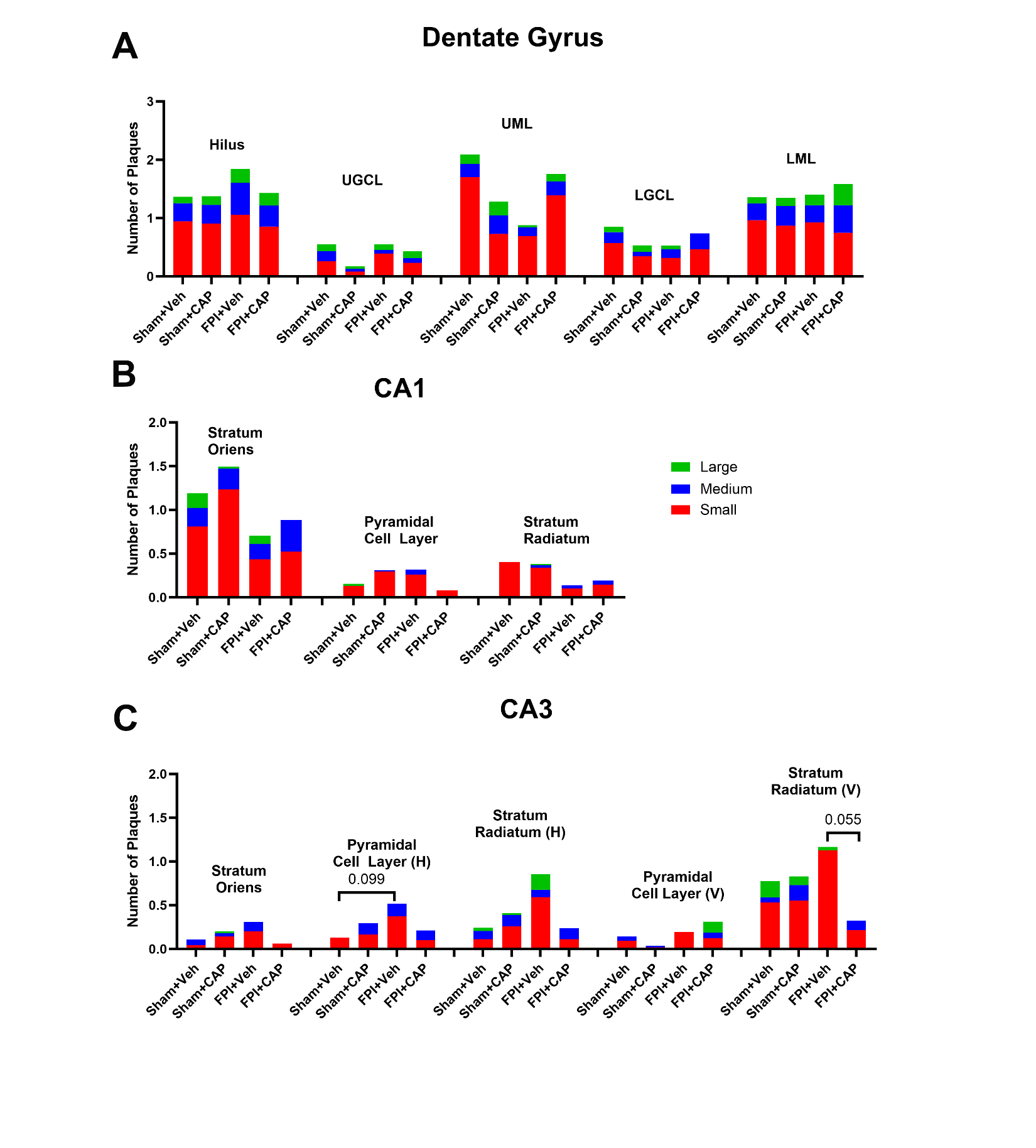


**Supplemental Figure 3. Plaque distribution by sublayer and size in the hippocampus.** In addition to total number, plaques were further analyzed by sub-layer and size in the dentate gyrus, CA1, and CA3. In A, there were limited effects of CAP and FPI on plaque distribution in the dentate. In **B,** FPI reduced plaques in CA1 stratum oriens and stratum radiatum but did not alter the size distribution. In **C,** FPI increased plaques in the CA3, most notably in the horizontal pyramidal cell layer and vertical stratum radiatum. FPI also appeared to increase the average size of plaques, so that there were more large plaques. Data are represented as Mean, stratified by plaque size.
